# Structural and mechanistic insights into the Artemis endonuclease and strategies for its inhibition

**DOI:** 10.1093/nar/gkab693

**Published:** 2021-08-13

**Authors:** Yuliana Yosaatmadja, Hannah T Baddock, Joseph A Newman, Marcin Bielinski, Angeline E Gavard, Shubhashish M M Mukhopadhyay, Adam A Dannerfjord, Christopher J Schofield, Peter J McHugh, Opher Gileadi

**Affiliations:** Centre for Medicines Discovery, University of Oxford, ORCRB, Roosevelt Drive, Oxford OX3 7DQ, UK; Department of Oncology, MRC-Weatherall Institute of Molecular Medicine, University of Oxford, Oxford OX3 9DS, UK; Centre for Medicines Discovery, University of Oxford, ORCRB, Roosevelt Drive, Oxford OX3 7DQ, UK; The Department of Chemistry and the Ineos Oxford Institute for Antimicrobial Research, Chemistry Research Laboratory, University of Oxford, Mansfield Road, Oxford OX1 3TA, UK; Centre for Medicines Discovery, University of Oxford, ORCRB, Roosevelt Drive, Oxford OX3 7DQ, UK; Centre for Medicines Discovery, University of Oxford, ORCRB, Roosevelt Drive, Oxford OX3 7DQ, UK; Centre for Medicines Discovery, University of Oxford, ORCRB, Roosevelt Drive, Oxford OX3 7DQ, UK; The Department of Chemistry and the Ineos Oxford Institute for Antimicrobial Research, Chemistry Research Laboratory, University of Oxford, Mansfield Road, Oxford OX1 3TA, UK; Department of Oncology, MRC-Weatherall Institute of Molecular Medicine, University of Oxford, Oxford OX3 9DS, UK; Centre for Medicines Discovery, University of Oxford, ORCRB, Roosevelt Drive, Oxford OX3 7DQ, UK

## Abstract

Artemis (SNM1C/DCLRE1C) is an endonuclease that plays a key role in development of B- and T-lymphocytes and in dsDNA break repair by non-homologous end-joining (NHEJ). Artemis is phosphorylated by DNA-PKcs and acts to open DNA hairpin intermediates generated during V(D)J and class-switch recombination. Artemis deficiency leads to congenital radiosensitive severe acquired immune deficiency (RS-SCID). Artemis belongs to a superfamily of nucleases containing metallo-β-lactamase (MBL) and β-CASP (CPSF-Artemis-SNM1-Pso2) domains. We present crystal structures of the catalytic domain of wildtype and variant forms of Artemis, including one causing RS-SCID Omenn syndrome. The catalytic domain of the Artemis has similar endonuclease activity to the phosphorylated full-length protein. Our structures help explain the predominantly endonucleolytic activity of Artemis, which contrasts with the predominantly exonuclease activity of the closely related SNM1A and SNM1B MBL fold nucleases. The structures reveal a second metal binding site in its β-CASP domain unique to Artemis, which is amenable to inhibition by compounds including ebselen. By combining our structural data with that from a recently reported Artemis structure, we were able model the interaction of Artemis with DNA substrates. The structures, including one of Artemis with the cephalosporin ceftriaxone, will help enable the rational development of selective SNM1 nuclease inhibitors.

## INTRODUCTION

Nucleases catalyse the hydrolysis of the phosphodiester bonds in nucleic acids and are broadly classified as exonucleases or endonucleases. Exonucleases are often non sequence-specific, while endonucleases can be further grouped into sequence-specific endonucleases, such as restriction enzymes, and structure-selective endonucleases (1). Artemis (SNM1C or DCLRE1C), along with SNM1A (DCLRE1A) and SNM1B (Apollo or DCLRE1B), are human nucleases that are members of the extended structural family of metallo-β-lactamase (MBL) fold enzymes (2,3). The N-terminal region of Artemis has a core MBL fold (aa 1–170, 319–361) with an inserted β-CASP (CPSF73, Artemis, SNM1 and PSO2) domain (aa 170–318) (4). β-CASP domains are present within the larger family of eukaryotic nucleic acid processing MBLs and confer both DNA/RNA binding and nuclease activity (2,5). The C-terminal region of Artemis (aa 362–692) mediates protein-protein interactions, contains post translational modification (PTM) sites, directs subcellular localization, and may modulate catalysis (6–9).

Although SNM1A, SNM1B and Artemis have similar structures of their core catalytic domains, each has distinct functions and selectivities. While SNM1A and SNM1B are exclusively 5′ to 3′ exonucleases, Artemis is an endonuclease (7,9), although a minor 5′ to 3′ exonuclease activity has been reported (10). Human SNM1A localizes to sites of DNA damage, can digest past DNA damage lesions *in vitro*, and is involved in inter-strand crosslink (ICL) repair (11–13). SNM1B is a shelterin-associated protein required for resection at newly-replicated leading-strand telomeres to generate the 3′-overhang necessary for telomere loop (t-loop) formation and telomere protection (14–16). SNM1A and SNM1B prefer ssDNA substrates *in vitro*, with an absolute requirement for a free 5′-phosphate (3,17). By contrast, Artemis preferentially cleaves hairpins and DNA junctions, although it is able to process ssDNA substrates (18–20).

The key roles of Artemis and related DSB repair enzymes in both programmed V(D)J recombination and non-programmed c-NHEJ DSB repair, make them attractive targets for treatment of cancer, either on their own or in combination with chemo- or radiotherapy. The Artemis endonuclease activity is responsible for hairpin opening in variable (diversity) joining (V(D)J) recombination (21) and contributes to end-processing in the canonical non-homologous end joining (c-NHEJ) DNA repair pathway (22–25). V(D)J recombination is initiated by recognition and binding of recombination-activating gene proteins (RAG1 and RAG2) to recombination signal sequences (RSSs) adjacent to the V, D and J gene segments. Upon binding, the RAG proteins induce double-strand breaks (DSBs) and create a hairpin at the coding ends (26–28). The Ku heterodimer recognizes the DNA double-strand break and recruits DNA-dependent protein kinase catalytic subunit (DNA-PKcs) and Artemis to mediate hairpin opening (19). Following hairpin opening, the NHEJ machinery containing the XRCC4/XLF(PAXX)/DNA-Ligase IV complex is recruited to catalyse the processing and ligation reactions at the DNA ends (22,29,30). V(D)J recombination is an essential process in antibody maturation (18,31,32).

Mutations in the Artemis gene cause aberrant hairpin opening, resulting in severe combined immune deficiency (RS-SCID), with sensitivity to ionizing radiation due to impairment of the predominant DSB repair pathway in mammalian cells, NHEJ (19,21,29), and another form of SCID (Omenn syndrome) associated with hypomorphic Artemis mutations (33,34). Artemis loss-of-function mutations often comprise large deletions in the first four exons or nonsense founder mutation, as found in Navajo and Apache Native Americans (35). In addition, missense mutations and in-frame deletions in the highly conserved residues such as H35, D165 and H228 can also abolish Artemis’ protein function (36).

We present high-resolution crystal structures of the catalytic core of Artemis (aa 1–361) containing both MBL and a β-CASP domains. The structures reveal that Artemis possesses a second metal binding site in its β-CASP domain, not present in SNM1A and SNM1B, that resembles classical Cys_2_His_2_ zinc finger motifs. Based on our data and another recently reported Artemis structure (37), we present a model for Artemis DNA binding. The model is compared with models of DNA binding from related nucleases, revealing distinct features that define a role for Artemis in the end-joining reaction. Following development of an assay suitable for screening, we identified β-lactam containing Artemis inhibitors, one of which, the cephalosporin ceftriaxone, was characterized crystallographically, providing information useful for structure-based design of inhibitors.

## MATERIALS AND METHODS

### Cloning and site directed mutagenesis of WT and mutant Artemis (aa 1-362)

Constructs encoding the Artemis MBL-β-CASP domain (WT and mutant) were cloned (using ligation independent cloning (LIC) (38)) into the baculovirus expression vector pBF-6HZB (GenBank™ accession number KP233213.1), which combines an N-terminal His_6_ sequence,the Z-basic tag and a TEV protease cleavage site for efficient purification. Site-directed mutagenesis was carried out using an inverse PCR method whereby an entire plasmid is amplified using complementary mutagenic primers (oligonucleotides) with minimal cloning steps (39), using the Herculase II Fusion DNA Polymerase (Agilent) for amplification and the KLD enzyme mix (NEB).

### Expression and purification of WT and mutant Artemis with IMAC (aa 1-362)

Baculovirus generation was performed as described (3). Recombinant proteins were produced by infecting *Sf9* cells at 2 × 10^6^ cells/ ml with 1.5 ml of P2 virus for WT and 3 ml of P2 virus for mutants respectively. Infected *Sf9* cells were harvested 70 h after infection by centrifugation (900 × g, 20 min). The cell pellets were resuspended in 30 ml/l lysis buffer (50 mM HEPES pH 7.5, 500 mM NaCl, 10 mM imidazole, 5% (v/v) glycerol and 1 mM TCEP), snap frozen in liquid nitrogen, then stored at −80°C for later use.

Thawed cell aliquots were lysed by sonication. The lysates were centrifuged (40 000 × g, 30 min); the supernatant was passed through a 0.80 μm filter (Millipore), then loaded onto an immobilized metal affinity chromatography column (IMAC) (Ni-NTA Superflow Cartridge, Qiagen) equilibrated in lysis buffer. The column was washed with lysis buffer, then eluted using a linear gradient to elution buffer (50 mM HEPES pH 7.5, 500 mM NaCl, 300 mM imidazole, 5% (v/v) glycerol and 1 mM TCEP). Protein-containing fractions were pooled and passed through an ion exchange column (HiTrap^®^ SP FF GE Healthcare Life Sciences) pre-equilibrated in the SP buffer A (25 mM HEPES pH 7.5, 300 mM NaCl, 5% (v/v) glycerol and 1 mM TCEP). Protein was eluted using a linear gradient to SP buffer B (SP buffer A with 1 M NaCl), and fractions containing the ZB-tagged Artemis were identified by electrophoresis.

Artemis containing fractions were pooled and dialysed overnight at 4°C in SP buffer A, supplemented with recombinant tobacco etch virus (TEV) protease to cleavage the His_6_-ZB tag. The protein was loaded into an ion exchange column (HiTrap^®^ SP FF GE Healthcare Life Sciences), pre-equilibrated in the SP buffer A. The protein was eluted using a linear gradient to SP buffer B; fractions containing tag-free Artemis were identified by electrophoresis. Artemis-containing fractions from the SP column elution were combined and concentrated to 1 ml using a 30 kDa MWCO centrifugal concentrator. The protein was then loaded on to a Superdex 75 increase 10/300 GL equilibrated with SEC buffer (25 mM HEPES pH 7.5, 300 mM NaCl, 5% (v/v) glycerol, 2 mM TCEP).

Mass spectrometric analysis of the purified proteins revealed masses of 41716.5, 41650.5, 41672.2, 41639.9 Da for WT, H35A, D37A and H35D proteins, respectively. The calculated masses are 41715.09, 41649.2, 41671.2 and 14639.2, respectively, all within 1.5 Da of the measured masses.

### Expression and purification of WT truncated Artemis catalytic domain without IMAC (aa 1–362)

The truncated Artemis protein was produced in a similar manner except that the first purification step (IMAC) was omitted. The tight binding of the ZB-tagged protein to the ion exchange column and the early elution of the tag-free protein allowed to achieve effective purification. After the second ion exchange step, the protein was further purified by size exclusion chromatography (Highload® 16/200 Superdex^®^ 200).

### Cloning, expression, and purification of full-length WT Artemis (aa 1-692)

The full-length Artemis encoding construct was cloned into pFB-CT10HF-LIC, a baculovirus transfer vector containing a C-terminal His_10_ and FLAG tag, using ligation independent cloning (LIC) (38). pFB-CT10HF-LIC was a gift from Nicola Burgess-Brown (Addgene plasmid # 39191; http://n2t.net/addgene:39191; RRID: Addgene_39191).

The baculovirus mediated expression of the full length DCLRE1C/ Artemis gene was performed in a manner similar to that for the truncated protein using 3.0 ml of P2 virus to infect *Sf9* cells at 2 × 10^6^ cells/ml.

Cell harvesting and the initial IMAC purification steps were performed as described for the catalytic domain. Following IMAC purification and TEV cleavage overnight in dialysis buffer (50 mM HEPES pH 7.5, 0.5 M NaCl, 5% glycerol and 1 mM TCEP) the protein was passed through a 5 ml Ni-sepharose column to trap the tag and other metal-binding contaminants; the flowthrough fractions were collected. The Artemis protein was then concentrated using a centrifugal concentrator (Centricon, MWCO 30 kDa) before loading on a Superdex S200 HR 16/60 gel filtration column in dialysis buffer. Fractions containing purified Artemis protein were pooled and concentrated to 10 mg/ml.

### Electrospray ionization mass spectrometry

Reversed-phase chromatography was performed prior to mass spectrometry using an Agilent 1290 uHPLC system (Agilent Technologies Inc., Palo Alto, CA, USA). Concentrated protein samples were diluted to 0.02 mg/ml in 0.1% aqueous formic acid and 50 μl was injected on to a 2.1 mm × 12.5 mm *Zorbax* 5um 300SB-C3 guard column housed in a column oven set at 40°C. The solvent system used consisted of 0.1% aqueous formic acid in ultra-high purity water (Millipore) (solvent A) and 0.1% aqueous formic acid in methanol (LC–MS grade, Chromasolve) (solvent B). Initial chromatography conditions were 90% A and 10% B and a flow rate of 1.0 ml/min. A linear gradient from 10% B to 80% B was applied over 35 s. Elution then proceeded isocratically at 95% B for 40 s followed by equilibration at initial conditions for a further 15 s. Protein intact mass was determined using a 6530-electrospray ionization quadrupole time-of-flight mass spectrometer (Agilent Technologies Inc., Palo Alto, CA, USA). The instrument was configured with the standard ESI source and operated in positive ion mode. The ion source was operated with the capillary voltage at 4000 V, nebulizer pressure at 60 psig, drying gas at 350°C and drying gas flow rate at 12 l/min. The instrument ion optic voltages were as follows: fragmentor 250 V, skimmer 60 V and octopole RF 250 V.

### Protein crystallization and soaking

Artemis (PDB: 6TT5) was crystallized using the sitting drop vapour diffusion method by mixing 50 nl protein with 50 nl crystallization solution comprising 0.2 M ammonium chloride, 20% (v/v) PEG 3350. Crystals appeared after 2 weeks and reached maximum size within 3 weeks. The crystals were soaked in cryoprotectant solution (mother liquor supplemented with 20% (v/v) ethylene glycol), then flash frozen in liquid nitrogen.

The non-IMAC purified Artemis (PDB: 7AF1) was crystallized in a similar manner, with addition of 20 nl of crystal seed solution obtained from previous crystallization experiment. Crystals were grown in a solution comprising 0.25 M ammonium chloride and 30% (v/v) PEG 3350 at 4°C. Crystals appeared after one day and reached a maximum size within one week.

Artemis variants (mutants H33A and H35D) were crystallized using the sitting drop vapour diffusion method by mixing 50 nl protein with 50 nl crystallization solution comprising 0.1 M sodium citrate pH 5.5, 20% PEG 3350; the D37A variant was crystalized in 0.2 M ammonium acetate, 0.1 M bis-Tris pH 5.5, 25% PEG 3350. All Artemis variants were crystalized in the presence of 20 nl of crystal seed solution obtained from previous crystallization experiment. Crystals grew after one day at 4°C. and reached maximum size within 1 week.

### Data collection and refinements

Data were collected at Diamond Light Source beamlines I04, I03 and I24. Diffraction data were processed using DIALS (40) and structures were solved by molecular replacement using PHASER (41) and the PDB coordinates 5Q2A. Model building and the addition of water molecules were performed in COOT (42) and structures refined using REFMAC (43). Data collection and refinement statistics are given in Table I. The X-ray fluorescence data was collected at Diamond Light Source I03 (6TT5) using 100% transmission and 12.7 eV, and I24 (7AF1) using 1% transmission and 12.8 eV (Supplementary Figure S1).

### Generation of 3′-radiolabelled substrates

10 pmol of single-stranded DNA (Eurofins MWG Operon, Germany) was labelled with 3.3 pmol of α-^32^P-dATP (Perkin Elmer) by incubation with terminal deoxynucleotidyl transferase (TdT, 20 U; ThermoFisher Scientific), at 37°C for 1 h. This solution was then passed through a P6 Micro Bio-Spin chromatography column (BioRad), and the radiolabeled DNA was annealed with the appropriate unlabeled oligonucleotides (1:1.5 molar ratio of labelled to unlabeled oligonucleotide) (Supplementary Table S1 for sequences) by heating to 95°C for 5 min and gradual cooling to below 30°C in annealing buffer (10 mM Tris–HCl; pH 7.5, 100 mM NaCl, 0.1 mM EDTA).

### Gel-based nuclease assays

Standard nuclease assays were carried out in reactions containing 20 mM HEPES–KOH, pH 7.5, 50 mM KCl, 10 mM MgCl_2_, 0.05% (v/v) Triton X-100, 5% (v/v) glycerol (final volume: 10 μl), and the indicated concentrations of Artemis. Reactions were started by addition of substrate (10 nM), incubated at 37°C for the indicated time, then quenched by addition of 10 μl stop solution (95% formamide, 10 mM EDTA, 0.25% (v/v) xylene cyanole, 0.25% (v/v) bromophenol blue) with incubation at 95°C for 3 min.

Reaction products were analysed by 20% denaturing polyacrylamide gel electrophoresis (made from 40% solution of 19:1 acrylamide:bis-acrylamide, BioRad) and 7 M urea) in 1× TBE (Tris–borate–EDTA) buffer. Electrophoresis was carried out at 700 V for 75 min; gels were subsequently fixed for 40 min in a 50% methanol, 10% acetic acid solution, and dried at 80°C for 2 h under a vacuum. Dried gels were exposed to a Kodak phosphor imager screen and scanned using a Typhoon 9500 instrument (GE).

### Fluorescence-based nuclease assay

The protocol of Lee *et al.* (44) was adapted for structure-specific endonuclease activity. A ssDNA substrate was utilized containing a 5′ FITC-conjugated T and a 3′ BHQ-1 (black hole quencher)-conjugated T (Supplementary Table S1). As the FITC and BHQ-1 are located proximal to one another, prior to endonucleolytic incision, the intact substrate does not fluoresce. Following endonucleolytic incision by SNM1C/Artemis, uncoupling of FITC from BHQ-1 causes an increase in fluorescence. Inhibitors (at increasing concentrations) were incubated with Artemis (50 nM) for 10 min at room temperature, before starting the reaction by adding the DNA substrate (25 nM). Assays were carried out in a 384-well format, in 25 μl reaction volumes. The buffer was the same as for the gel-based nuclease assays., Fluorescence spectra were measured using a PHERAstar FSX (excitation: 495 nm; emission: 525 nm) with readings taken every 150 s, for 35 min, at 37°C.

## RESULTS

### Human Artemis (SNM1C or DCLRE1C) has a core catalytic fold similar to SNM1A and Apollo/SNM1B

**Figure 1.**
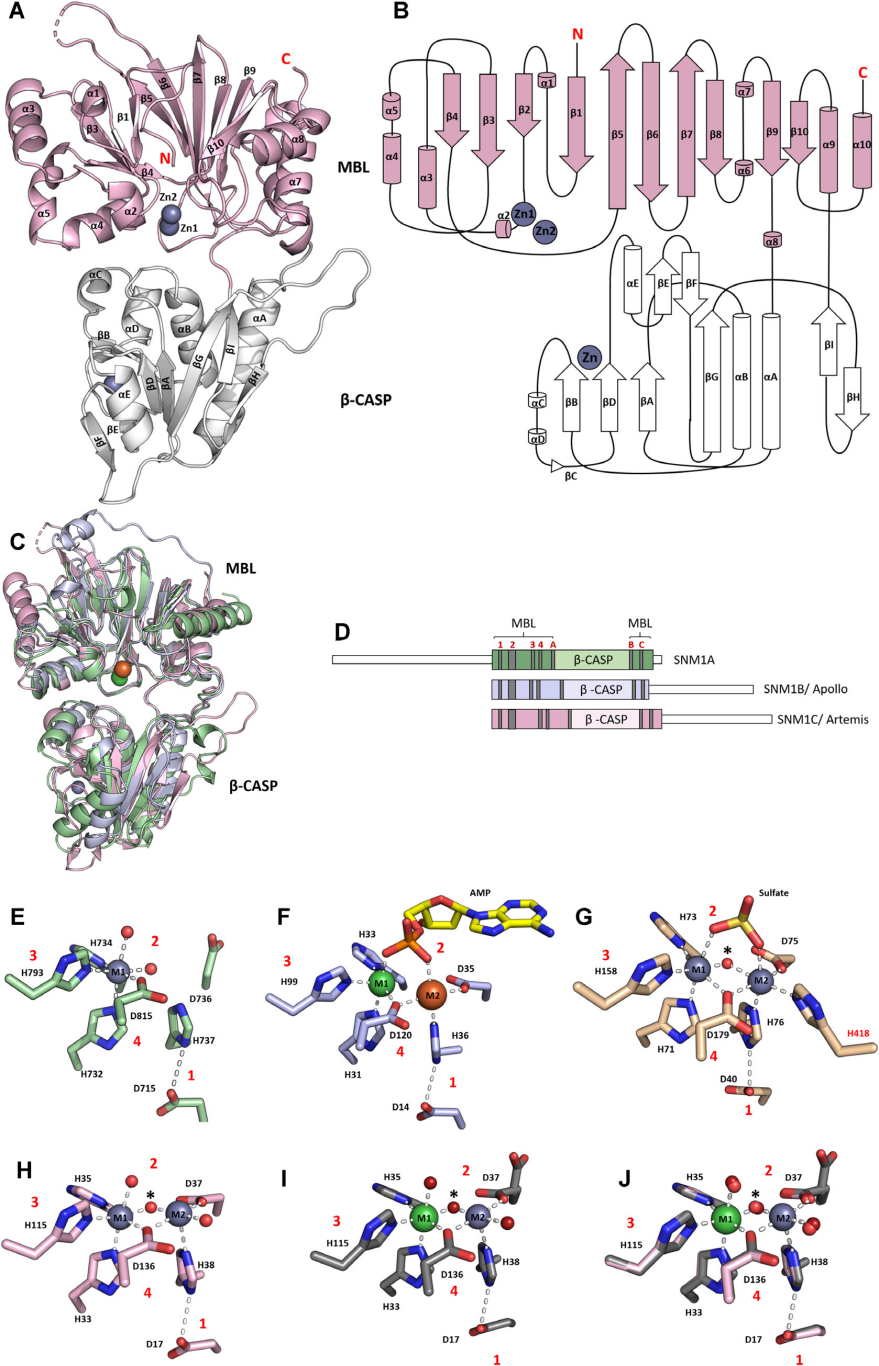
Overall fold of the SNM family and the active site views of human MBL/β-CASP nuclease fold enzymes. (**A**) Cartoon representation of the structure of human SNM1C/ Artemis; the active site containing MBL domain in pink; the β-CASP domain (white) contains a novel zinc-finger like motif. The three zinc ions are represented by grey spheres; the N- and C-termini are marked in red lettering. (**B**) Topology of Artemis; β-strands are represented as arrows and α-helices as cylinders. The MBL domain (pink) has the typical α/β-β/α sandwich fold, with the β-CASP domain (white) inserted between the small helix α8 and α9. (**C**) Overlay of structures of the human SNM1A, SNM1B and SNM1C. (**D**) Cartoon sequence alignment for SNM1A, SNM1B and Artemis, showing the MBL and β-CASP domains. Each of the 4 highly conserved motifs (1–4), are in red. Motif 1 = Asp, motif 2 = 3 His and 1 Asp (HxHxDH), motif 3 = His and motif 4 = Asp. (**E**) SNM1A as purified has a single octahedrally coordinated zinc (grey) (PDB: 5AHR). (**F**) Active site view of SNM1B/ Apollo (PDB: 7A1F) with a nickel ion (green, M1) and an iron ion (orange, M2) with a coordinating AMP molecule. (**G**) Active site view of the human RNA processing enzyme CPSF73 (PDB: 2I7V) containing a sulphate ion and a water bridging (asterisk*) the two zincs. One zinc (M2) is coordinated by an additional histidine residue (His 418) which has no counterpart in SNM1 proteins. (**H**) Artemis (PDB: 7AF1) purified in the absence of IMAC has two zincs (grey) in its active site. A water/hydroxide shared (asterisk*) between the two metals is the proposed nucleophile for the hydrolytic reaction. (**I**) Active site of human Artemis (PDB: 6TT5) purified with IMAC. A nickel is present in the first metal coordination site (M1) and a zinc in the second (M2). (**J**) The overlay of Artemis active site views (H) (PDB: 7AF1) and (I) (PDB: 6TT5).

The catalytic domain of Artemis (aa 3–361) fused to the basic His_6_-Zb tag, which confers tight binding to cation exchange columns, was produced in baculovirus-infected *Sf9* cells. The protein was purified using immobilized metal affinity chromatography (IMAC) on a nickel-Sepharose column as the initial step. Subsequent preparations were performed without the use of IMAC, to avoid introduction of Ni^2+^ ions. Artemis crystals were grown and diffracted to 1.6 Å resolution (Table 1); the structure was solved using a structure of SNM1A (PDB: 5Q2A) as a molecular replacement model. The resultant structure (PDB: 7AF1; space group P1) contains a single molecule in the asymmetric unit, with two active site zinc ions (i.e. at both M1 and M2 sites) (Figure 1). Copurifying metal ions were identified using X-ray fluorescence (XRF) analysis. With the protein purified using IMAC, the structure was refined with a full occupancy nickel ion at the M1 site and a partial occupancy zinc ion at the M2 site (PDB: 6TT5) (Figure 1I); the identification of a nickel ion (at M1 and/or M2 sites) was supported by XRF (Supplementary Figure S1). A similar overall metal ion coordination pattern has been observed with other members of the family, such as SNM1A and SNM1B/Apollo (3,11).

**Table 1.** Data collections and refinement statistics

PDB ID	6TT5 (Ni and Zn)	7AF1 (2 Zn)	7AFS (D37A)	7AFU (H33A)	7AGI (H35D)	7APV (Ceftriaxone)	7ABS (DNA bound)
**Data Collection and processing**							
Diffraction Source	DLS (I04)	DLS (I24)	DLS (I03)	DLS (I03)	DLS (I03)	DLS (I03)	APS 17-IDD
Wavelength (Å)	0.979	0.976	0.976	0.976	0.976	0.976	1.00
Space group	*P*1	*P*1	*P*1	*P*1	*P*1	*P*1	*P*21212
Cell dimensions							
*a, b, c* (Å)	35.87, 47.99, 48.25	35.75, 47.97, 48.15	35.91, 48.06, 48.21	35.97, 47.90, 48.37	35.97, 48.05, 48.44	35.88, 48.10, 48.25	72.81, 111.00, 55.17
*α, β, γ* (°)	82.61, 76.37, 85.98	82.68, 76.35, 85.81	82.89, 76.43, 86.38	82.51, 75.94, 87.73	82.43,76.01, 87.33	82.76, 76.29, 86.30	90.00, 90.00, 90.00
Resolution (Å) *	47.55–1.50	47.53–1.70	35.35–1.70	35.53–1.56	47.62–1.70	47.69–1.95	34.5–1.97
	(1.53–1.50)	(1.73–1.70)	(1.73–1.70)	(1.59–1.56)	(1.73–1.70)	(2.00–1.95)	(2.02–1.97)
*R*_merge_ (%)*	6.0 (64.4)	13.3 (79.5)	5.3 (53.2)	4.9 (31.5)	4.8 (23.2)	11.6 (65.0)	5.0 (82.8)
*I/σ*(*I*)	13.4 (3.2)	5.8 (2.0)	11.7 (2.2)	11.3 (2.6)	13.4 (3.8)	8.6 (2.8)	16 (2.4)
Completeness (%)	94.7 (66.4)	97.4 (95.6)	97.3 (96.0)	94.2 (63.7)	97.4 (95.4)	98.0 (97.2)	99.6 (99.6)
Multiplicity	3.6 (3.2)	3.5 (3.5)	3.6 (3.7)	3.6 (3.3)	3.7 (3.8)	3.5 (3.6)	6.4 (6.6)
**Refinements**							
Resolution (Å)	47.55–1.50	47.53–1.70	47.66–1.70	35.53–1.56	47.62–1.70	47.96–1.95	40–1.97
No. of reflections	44 746	31 282	34 470	39 478	31 745	21 086	30 565
*R*_work_	0.17	0.19	0.18	0.19	0.19	0.18	0.22
*R*_free_	0.19	0.21	0.22	0.21	0.21	0.23	0.28
No. of atoms							
Protein	2927	2989	2957	2985	3122	2975	2923
Water	224	144	163	255	164	107	105
Zinc/nickle	3	3	2	1	1	2	3
Ethylene glycol	24	28	12	44	32	16	–
DNA	–	–	–	–	–	–	253
Ceftriaxone	–		–	–	–	1	–
*B*-factors	16.3	19.3	25.9	17.5	18.4	22.6	56
r.m.s. deviations							
Bond length (Å)	0.003	0.008	0.008	0.006	0.007	0.01	0.007
Bond angles (°)	1.23	1.33	1.29	1.28	1.32	1.5	1.47

* Data in parentheses is for the high-resolution shell.

The overall fold of the catalytic core of Artemis is similar to that of SNM1A and SNM1B (2 Å backbone RMSD); it has all the key structural characteristics of human MBL fold nucleases, with the di-metal containing active site at the interface between the MBL and β-CASP domains (Figure 1A). Its MBL domain (Figure 1A, B) has the typical α/β-β/α sandwich MBL fold (45) and contains the conserved motifs 1–4 (Figure 1C and D, Supplementary Figure S2) typical of the MBL superfamily and motifs A–C typical of the β-CASP fold family (2,4,29,46).

Motifs 1–4 (Figure 1D–J) are responsible for metal ion coordination in both DNA and RNA processing MBL fold enzymes (4). As observed in structures of SNM1A and SNM1B, Artemis can coordinate one or two metal ions in its active site. In the di-zinc complexed structure, one zinc ion (M1) is coordinated by four residues (His33, His35, His115 and Asp116) and two water molecules (H_2_O 506 and 611) in an octahedral manner (Figure 1H). The second zinc ion (M2) was refined with 30% occupancy and is coordinated by Asp37, His38 and Asp136 and two waters. The low occupancy of the second zinc ion, together with the two conformations (0.5 occupancy for each conformation) observed for Asp37 (Figure 1I and J) suggest that the M2 site binds a metal ion less tightly than the M1 site, consistent with studies on other human MBL fold nucleases (3, Baddock *et al.* 2021)

The structure of human SNM1A has been solved with a single zinc ion coordinated in its active site (Figure 1E) (3). By contrast, SNM1B structures solved with a bound AMP [Baddock *et al.*, 2021 accompanying paper] show two metal ions, both positioned to coordinate the phosphate group of the AMP (Figure 1F). In summary, the coordination for the M1 site zinc ion involves three histidines, one aspartate, and either water molecules or a phosphate oxygen of the substrate. The M2 site metal ion is more weakly coordinated in the SNM1 family, with one histidine and two aspartates, with the remaining three positions occupied by water or a phosphate oxygen of the substrate. Thus, the presence of the substrate at the active site may help promote full metal ion occupancy at the M2 site. We propose that Artemis coordinates a phosphate group of its substrate in a similar manner to that proposed for SNM1B.

A structure of human CPSF-73 (PDB: 2I7V), an MBL RNA processing nuclease, has been solved with two active site bound zinc ions, an adjacent sulphate, and a bridging water molecule proposed to play an important role in hydrolysis (Figure 1G) (47). The CPSF-73 structure shows that the two zinc ions are coordinated in a very similar geometry with the human MBL DNA processing enzymes (48,49). A striking difference between the MBL RNA and DNA nucleases is that the second metal ion (M2) in the RNA processing nucleases is coordinated by an additional histidine (His418 for CPSF-73) (47), which has no counterpart in the DNA processing enzymes.

### The structure of Artemis reveals a novel zinc-finger like motif in the β-CASP domain

**Figure 2.**
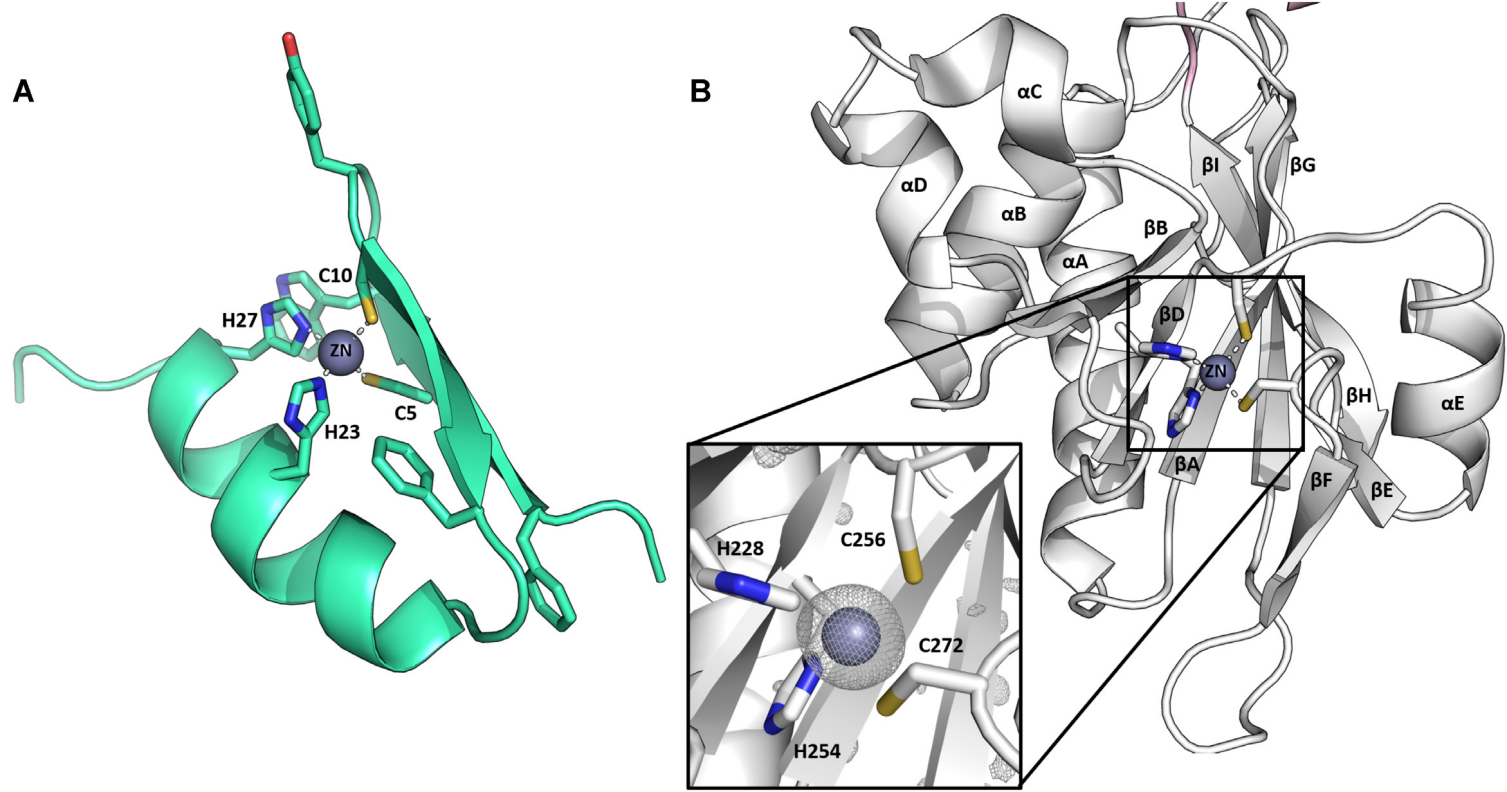
Comparison of a novel zinc-finger like motif in the β-CASP domain of SNM1C/Artemis with a canonical zinc-finger motif. (**A**) Cartoon representation of a classical Cys_2_His_2_ zinc-finger motif (green) from the transcription factor SP1F2 (PDB code: 1SP2). This has a ββα fold, where two Cys- and two His-residues are involved in zinc ion coordination and the sidechains of three conserved hydrophobic residues are shown. (**B**) The β-CASP region of Artemis has a novel zinc-finger like motif. The inset shows the four residues (two His and two Cys) coordinating the zinc ion (grey). The *F*_o_ – *F*_c_ electron- density map (scaled to 2.5σ in PyMOL) surrounding the zinc ion before it was included in refinement is shown.

Proteins with a β-CASP fold form a distinct sub-group in the MBL- superfamily that specifically act on nucleic acids (2). Artemis’ β-CASP domain (aa 156–384) is inserted within the MBL fold sequence between α-helices 6 and 7 (Figure 1). Notably, a second metal ion binding site, unique to Artemis, with similarity to the canonical Cys_2_ His_2_ zinc-finger motif, is present in its β-CASP domain (50,51). Many DNA binding proteins, including transcription factors and DNA repair factors (including those involved in NHEJ), possess a Cys_2_ His_2_ zinc finger motif that stabilizes the DNA binding domain (50–53). A typical Cys_2_ His_2_ zinc coordinating finger (Figure 2A) has a ββα motif, wherein the zinc ion is coordinated between an α-helix and two antiparallel β-sheets. Hydrophobic residues located at the sides of the zinc coordination site enable specific binding of the zinc finger in the major groove of the DNA (50,51,54,55). Similar to the canonical Cys_2_ His_2_ zinc finger motif, the zinc ion coordination in Artemis’ β-CASP domain adopts a tetrahedral geometry, with coordination by two cysteine (Cys256, Cys272) and two histidine (His228, His254) residues (Figure 2B). However, in the case of Artemis the metal ion coordination site is sandwiched between two β-sheets instead of an α-helix and two antiparallel β-sheets.

All but one of the residues in the zinc-finger-like motif (His228, Cys256 and Cys227) are distinct to Artemis relative to SNM1A/B (Supplementary Figure S2), with only His254 seen in all three enzymes. However, the four residues that form the zinc-finger like motif are conserved in Artemis across the animal kingdom (from humans to sponges), implying functional importance (Supplementary Figure S3). Consistently, substitution of His228 and His254 (H228N and H254L), two of the zinc coordinating residues in the β-CASP domain of Artemis, cause RS-SCID in humans (4,36,56). Patients with these inherited mutations suffer from impaired V(D)J recombination, leading to underdeveloped B and T lymphocytes. The full-length H254A Artemis variant is unable to carry out V(D)J recombination *in vivo* and has no discernible endonucleolytic activity *in vitro* (4).

### Comparison of Artemis structures leading to a model for DNA binding

**Figure 3.**
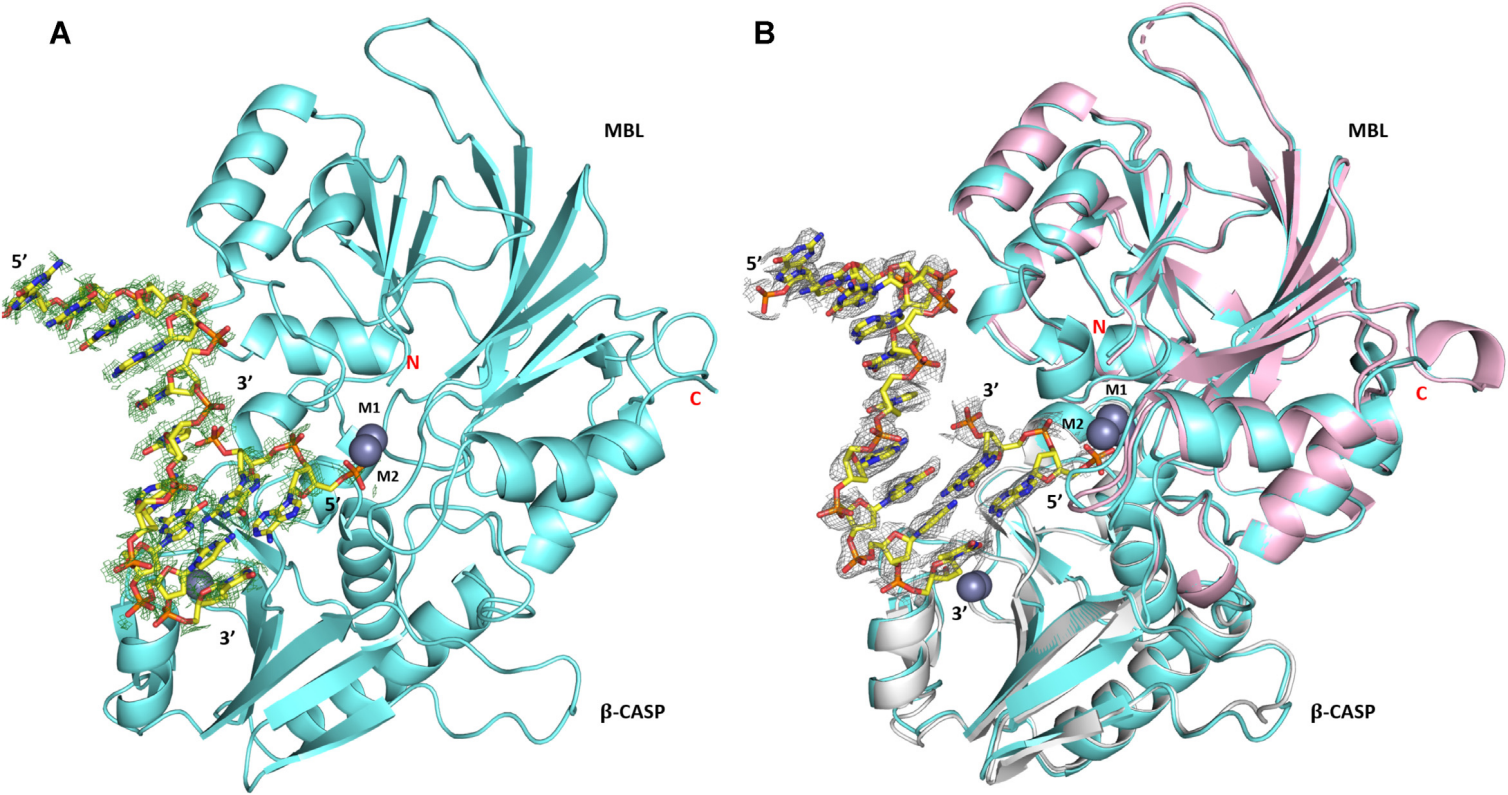
Electron density of a DNA molecule in previously reported structures of Artemis. (**A**) Analysis of the reported (PDB: 6WO0) Artemis structure re-refined with DNA present (PDB code: 7ABS). The *F*_o_*– F*_c_ electron density map (contoured at 0.6 σ in pymol) for the DNA is in green mesh. (**B**) The 7ABS structure (aquamarine) overlayed with the apo-Artemis structure (PDB code: 7AF1) in pink (backbone RMSD 0.48 Å). The 2*F*_o_*– F*_c_ electron density map (contoured at 0.6 σ in pymol) for the DNA is in grey mesh.

During our work two Artemis structures (PDB: 6WO0 and 6WNL) similar to our structure (PDB: 7AF1) (backbone RMSDs of 0.48 and 0.54 Å, respectively) were reported (37), with identical relative positioning of the MBL and β-CASP domains (Figure 3A and Supplementary Figure S4A). The only significant difference was that whilst we refined our structure PDB: 7AF1 with two zinc ions in the active site, both of the crystal forms reported by Karim *et al.* (37) were modelled with a single active site zinc ion (Zn1), reinforcing the proposal of weaker metal ion binding at the Zn2/M2 site.

A striking aspect of the reported (37) structures is that both crystal forms were obtained in the presence of DNA and were reported to require DNA for their growth; the crystals showed a fluorescence signal supporting the presence of DNA (the oligonucleotides used contained a cyanine dye fluorophore), yet neither of the models presented contain DNA. The authors referred to some broken stacking electron density in 6WNL in a solvent channel and a patch of unsolved density approaching the active site in 6WO0, but state that the DNA ‘did not bind to the protein in a physiological way, and likely bound promiscuously to promote crystallization’ (37). We re-examined these structures looking closely at the residual electron density. For the 6WNL structure we found evidence for a distorted duplex DNA of around 13 base pairs which we propose may be the product of duplex annealing of the oligonucleotide used for crystallization (a semi-palindromic 13-mer that was designed to form a hairpin with phospho-thioate linkages in the single-stranded region) (Supplementary Figure S4B). For this structure, we are in general agreement with Karim *et al.* that the DNA does not appear to make meaningful interactions with the protein that inform on the mechanism of nuclease activity, although this mode of association with DNA may be relevant to alternative binding modes relating to higher order complexes containing Artemis.

For the PDB: 6WO0 structure we were able to confidently build a DNA molecule that contacts the Artemis active site in a manner that we believe to be relevant to the Artemis nuclease activity. The model contains an 8-nucleotide 5′-single-stranded extension with a short 2-base pair region of duplex DNA that reaches into the Artemis active site, making close contacts with the metal ion centre in a manner consistent with the proposed catalytic mechanism (Figure 3). The sequence of the longest strand corresponds to the 10-nucleotide cy-5 labelled strand (cy5-GCGATCAGCT) with residual density at the 5′-end that may be attributed to the cyanine fluorophore which we did not include in our model. The complementary strand used in crystallization was 13-nucleotides long and was intended to produce a 5′-overhang, but only two bases and three phosphates could be located in the density. The abrupt manner in which the electron density apparently disappears from either end of this strand suggests that this is the product of a cleavage reaction, although it is possible that remaining nucleotides are not located due to disorder.

The analysis of electron density at this site is complicated by the proximity to a crystallographic 2-fold symmetry axis, which brings a symmetry copy of the DNA molecule into a position where atoms partially overlap and the extended 5′ strands form a pseudo duplex (Supplementary Figure S5A). The occupancy of the entire DNA molecule is thus limited to 0.5, and the lower occupancy is reflected in the electron density map which requires a lower contour level than would usually be applied (Supplementary Figure S5B). After carefully building and refining the afore-described DNA bound model, significant positive electron density was revealed for the second metal ion (M2 site) which we included in the model with the same occupancy (0.5) as the DNA. Our model was refined to similar crystallographic R-factors as 6WO0 and has been deposited with PDB accession number 7ABS (refinement statistics are given in Table I).

**Figure 4.**
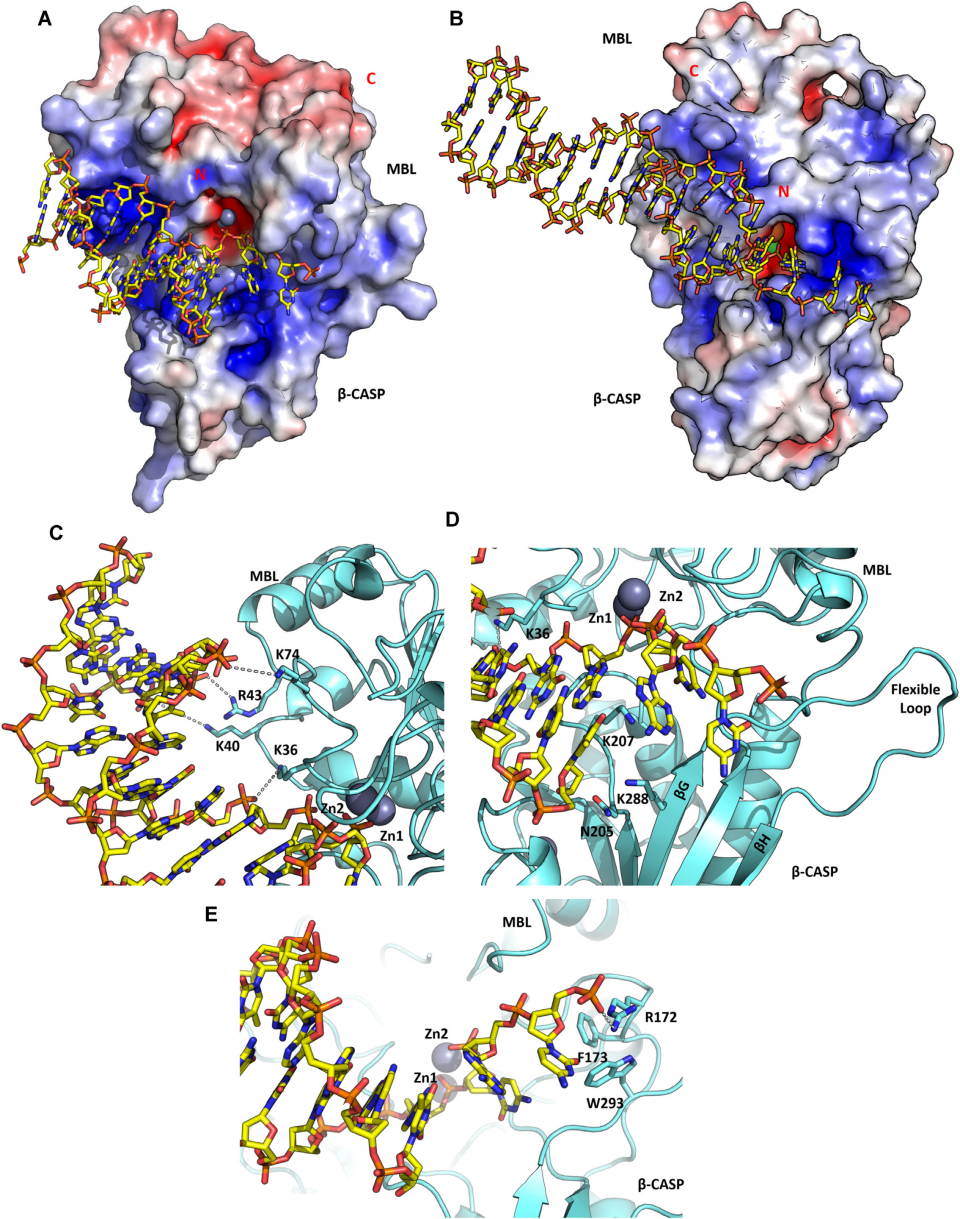
Proposed interactions of DNA with SNM1C/Artemis. Comparison of electrostatic surface potentials of DNA bound model for (**A**). Artemis and (**B**). Apollo/SNM1B [Baddock *et al.* 2021 accompanying paper]. Blue colouring represents a more electropositive surface potential and red show a more electronegative cluster. The active site contains two metal ions represented as a grey (zinc), orange (iron) and green (nickel) spheres. N- and C- termini are in red. Electrostatic surface potentials were generated using PyMOL (electrostatic range: ±5). (**C**) A row of positively charged residues is on the MBL domain surface of interact with the DNA phosphate backbone. (**D**) A DNA overhang is located at the active site. A cluster of polar residues (N205, K207 and K288) is located in the β-CASP domain. The extended flexible loop, which is unique to Artemis compared to SNM1A and SNM1B, connecting βG and βH is labelled. (**E**) The DNA overhang forms a hydrogen bond with Arg172 and interacts with a cluster of hydrophobic residues at the interface of the MBL and β-CASP domains.

Using the crystallographically observed DNA as a template we were able make a model for Artemis binding to a longer section of double-stranded DNA by complementing unpaired bases on the single-stranded DNA overhang with canonical base pairs, whilst maintaining acceptable geometry of the sugar phosphate backbone (Figure 4A). The duplex section of this model deviates slightly from the ideal B-form geometry (57), in a manner that is reminiscent of some transcription factor DNA complexes (58,59). We have also modelled an extension of the metal ion contacting strand by three nucleotides to form a 5′-overhang; the positioning of the overhang nucleotides is more speculative, nevertheless it was possible to avoid clashes with residues whist maintaining a relaxed geometry (Figure 4E).

In the extended DNA complex model, Artemis contacts both strands of the DNA in several areas; notably a single phosphate lies above the di-metal ion bearing active site and ligates to both metal ions in the same manner as in structures of related SNM1 enzymes (Baddock *et al.* 2021, accompanying paper) (60). The two downstream nucleotides on this strand pass close to the protein surface, forming possible interactions with the backbone NH of Asp37 and the sidechain of Lys36; subsequent nucleotides are not close to the protein (Figure 4C). The overhang portion of this strand continues with a slightly altered trajectory, potentially contacting Artemis in the vicinity of the cleft separating the MBL and β-CASP domains, with the possibility of forming favourable interactions with both positively charged (Arg 172) and aromatic residues (Phe173, Trp293) (Figure 4E). The surface between the MBL and β-CASP domains of Artemis contains a belt of positively charged residues (Figure 4A), which likely facilitates productive DNA binding.

The complementary strand interacts with the protein *via* backbone contacts that span a 4-nucleotide sequence between 5- and 8-bases from the 3′-terminus with positively charged sidechains in the MBL domain (Lys36, Lys40, Arg43 and Lys74) (Figure 4C). The 3′-end of this strand apparently terminates directly above a cluster of polar or positively charged residues in the β-CASP domain (Lys207, Lys288, Asn205) (Figure 4D). Whilst the experimental (as used in co-crystallization) DNA substrate and our model both contain a 3′-hydroxyl group, the model implies that an additional 3′-phosphate could be accommodated and may be expected to make favourable interactions with the basic cluster of residues. Thus, our model suggests Artemis preferentially binds DNA with a 5′-overhang binding at the junction between double- and single-stranded regions; the expected product of this reaction would be a blunt ended DNA with a 5′-phosphate. In the case of hairpin DNA substrates our model indicates Artemis may accommodate a 4-nucleotide loop connecting the two paired strands, with the cleavage product being DNA with a 3′-overhang cleaved from the last paired base of the duplex.

### Comparison of the Artemis DNA binding mode with that of other nucleases

In an accompanying paper [Baddock *et al.* 2021] we report a structure of SNM1B/Apollo in complex with two deoxyadenosine monophosphate nucleotides and suggest a model for SNM1B binding to DNA containing a 3′-overhang (one of its preferred substrates). The overall mode of DNA binding is similar in the two models (Figure 4A and B), with the two DNA duplexes being roughly parallel and forming contacts to similar regions on the MBL domain. The most important differences lie in the nature of the contacts formed to the active site and the paths of the various overhangs. In the SNM1B model, extensive contacts are made to the 5′-phosphate in a well-defined phosphate binding pocket. Both SNM1A and SNM1B are exclusively 5′-phosphate exonucleases, with most of these phosphate binding residues being conserved (Supplementary Figure S2). Artemis lacks these key phosphate binding residues and the 5′-phosphate binding pocket of SNM1A and SNM1B. Instead, in Artemis this pocket is partially filled by the Phe318 sidechain, which may inhibit the binding of the substrate 5′-phosphate. These differences rationalize major differences in nuclease activities within the family, i.e. SNM1A and SNM1B are exonucleases and Artemis is an endonuclease.

Further differences between Artemis and SNM1B/SNM1A are found in the loop connecting β-strands G and H (using Artemis numbering), which in Artemis is significantly longer and occupies a different position contacting residues in the MBL domain (Figure 4D), compared to the loops in SNM1B and SNM1A that form part of the phosphate binding pocket and make potential contacts with the 3′-overhang. This loop displacement in Artemis may contribute to its ability to accommodate substrates with either 5′-overhangs or hairpins.

Another striking difference, at least in the available structures, is that the active site of Artemis is more open compared to those of SNM1A or SNM1B. This openness may reflect an ability to accommodate different substrate conformations including hairpins, 3′- and 5′-overhangs, as well as DNA flaps and gaps. Both human SNM1A and SNM1B appear to have a more sequestered active site that would only fit a single strand of DNA, which is consistent with previous findings on their preferred substrate selectivity (10).

### Biochemical characterization of truncated Artemis catalytic domain (aa 1–361)

To investigate the activity of different versions of Artemis, we performed assays using radiolabelled DNA. We compared the catalytic domain purified using IMAC (which contained Ni^2+^ in the active site) with catalytic domain protein purified using ion exchange chromatography (avoiding IMAC), which contained predominantly Zn^2+^. We also tested the activity of full-length phosphorylated Artemis. The results show that both truncated enzymes have similar activity to the full-length enzyme (Supplementary Figure S6).

One notable difference between our full-length protein and that of Ma *et al.* (20), is that our protein is active in the absence of DNA-PKcs. Intact mass spectrometric analysis of full-length protein shows that it has undergone up to five phosphorylations (Supplementary Figure S7). Poinsignon *et al.* have shown that Artemis is constitutively phosphorylated in cultured mammalian cells and is further phosphorylated in response to induced DNA damage (61); it is interesting that the capacity to phosphorylate Artemis to produce an active form is also conserved in insect cells. We observed exonuclease activity with full-length Artemis at 10 nM enzyme concentration (Supplementary Figure S8), though this was weak compared to its endonuclease activity at the same concentration. We observed no exonuclease activity for the truncated Artemis construct, it is possible that phosphorylation alters the balance between endonuclease and exonuclease activity, though the biological relevance of this, if any, remains to be validated.

**Figure 5.**
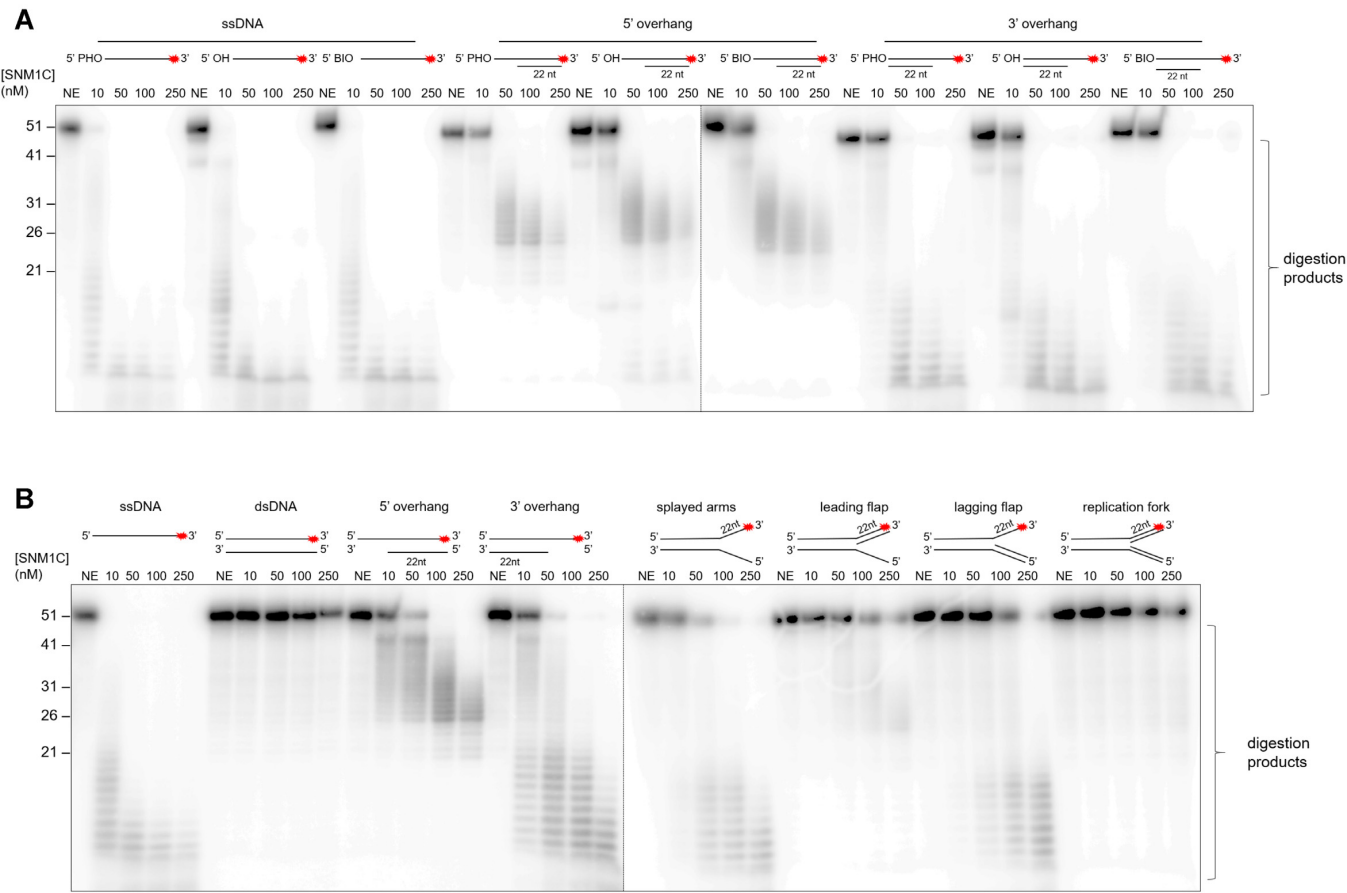
Nuclease assay of truncated Artemis (aa 3–361) with various DNA substrates. (**A**) The nuclease activity of Artemis is indifferent to the 5′ group, indicative of its endonuclease activity. Increasing concentrations of Artemis from 0 (NE; no enzyme) to 250 nM incubated with 10 nM ssDNA with either a 5′ phosphate, 5′ hydroxyl, or 5′ biotin moiety (45 min, 37°C). (**B**) Artemis cleaves DNA substrates containing single-stranded regions. Increasing amounts of Artemis were incubated with structurally diverse DNA substrates (10 nM) (45 min, 37°C). Products were analysed by 20% denaturing PAGE. The DNA substrates utilized are represented at the top of the lanes and a red asterisk indicates the position of the 3′ radiolabel.

Both human SNM1A and SNM1B require a 5′-phosphate for activity (3,11,17). To investigate whether there is a similar requirement for Artemis, we tested the activity of truncated Artemis against single-stranded and overhang DNA substrates with different 5′-end groups, including a phosphate, hydroxyl and biotin groups (Figure 5A). The results obtained imply that, at least under the tested conditions, Artemis is agnostic to the different end modifications, exhibiting comparable digestion of all substrates.

Extensive evidence demonstrates that full-length Artemis in complex with DNA-PKcs is a structure-selective endonuclease (19,20,29). These studies report Artemis can digest substrates including overhangs, hairpins, stem-loops, and splayed arms (pseudo-Y). To investigate the activity of truncated Artemis catalytic domain (aa 1–361) we performed assays using a variety of radio-labelled DNA substrates (Figure 5B). The results show that truncated Artemis has substrate-selective endonuclease activity, with a preference for single-stranded DNA substrates, and those that contain single stranded character (e.g. 5′- and 3′-overhangs, splayed arms, and a lagging flap structure), compared with double-stranded DNA structures (e.g. ds DNA and a replication fork). This is in accord with previous reports, where Artemis is reported to cleave around ss- to dsDNA junctions in DNA substrates (62). The truncated Artemis catalytic domain also exhibits hairpin opening activity, in accordance with what has previously been reported (Supplementary Figure S9). On a duplex substrate (YM117 from Ma *et al.*) (20) with a 20 nt hairpin region, Artemis cleaves adjacent to the hairpin, consistent with previous data. It is clear that truncated form of Artemis exhibits nuclease activity closely comparable to the phosphorylated full-length Artemis protein (20), indicating that the structural studies presented here reveal mechanistic insights of direct relevance to the DNA-PKcs-associated form of Artemis that engages in end-processing reactions *in vivo*.

### Structural and biochemical characterization of Artemis point mutations

**Figure 6.**
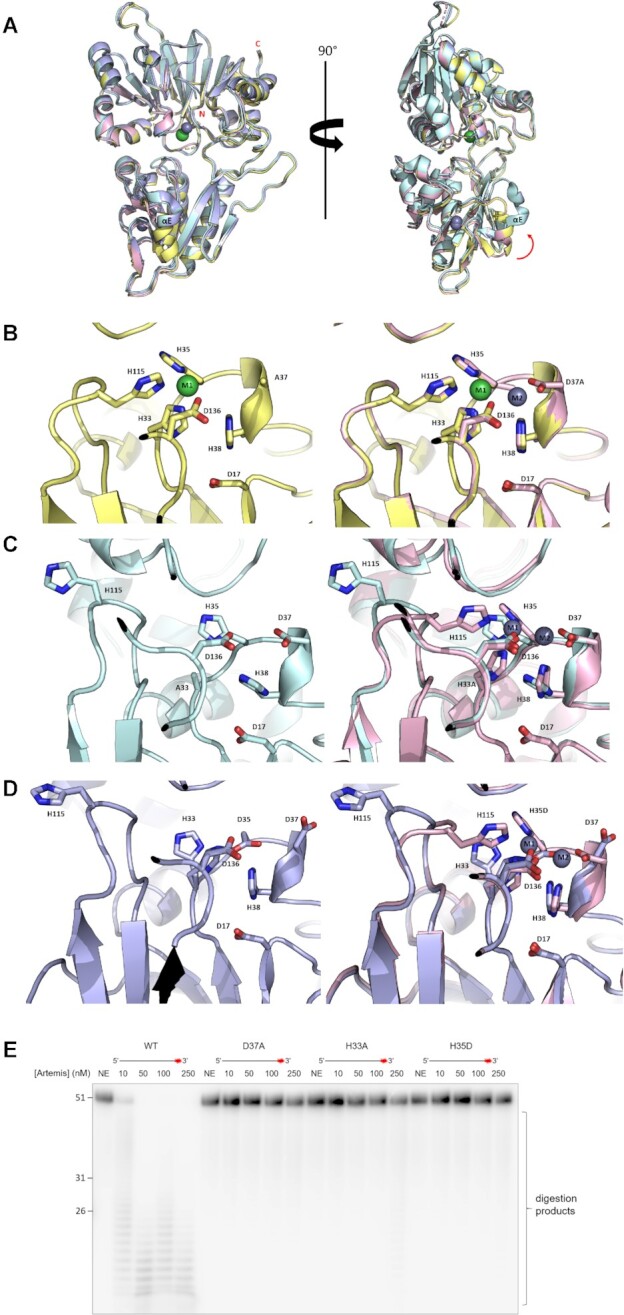
Structures of SNM1C/Artemis D37A, H33A and H35D variants and their activities. (**A**) Overlay of WT Artemis (PDB:7AF1, pink), and the D37A (PDB:7AFS, yellow), H33A (PDB:7AFU, cyan) and H35D clinical (in Omen syndrome) (PDB:7AGI, blue) variants showing the overall folds are conserved. The nickel ion is a green sphere and zinc ions are grey spheres. Movement of αE in the β-CASP domain is indicated by a red arrow. (**B**) Left: The active site of D37A variant has a single nickel ion (M1 in green); Right: active site residues of WT Artemis (pink), superimposed with that of the D37A variant (yellow)—aside from loss of the second metal (M2) in the latter, there is little difference. (**C**) Active site of the H33A variant (cyan; left) and an overlay (right) with WT Artemis (pink). The H33A variant is characterized by a lack of metal ions and a different conformation of the loop containing His115. (**D**) The active site of the H35D variant (blue; left) and an overlay (right) with WT Artemis (pink). These variant lacks both metal ions, as for the H33A variant. E. Comparing the activity of Artemis variants vs WT protein. Increasing amounts (from 0 to 250 nM) of WT and mutant Artemis proteins (were incubated with 10 nM of 51 nucleotide ssDNA substrate (30 min, 37°C). Products were analysed by 20% denaturing PAGE.

Previous mutagenesis studies targeting metal ion coordinating residues (D17N, H33A, H35A, D37N) of full-length Artemis (aa 1–692) established the importance of motifs 1–4 for activity (29). Each of these substitutions markedly reduced or abolished Artemis’ ability to carry out its role in V(D)J recombination *in vitro*. We crystallized three forms of truncated Artemis (aa 1–361) with substitutions in several of these metal ion co-ordinating residues, i.e. D37A, H33A, and the Omenn Syndrome patient mutation H35D (33,56). The overall architecture of the three variants is almost identical to WT Artemis (Figure 6A). The D37A structure crystallizes with only one nickel ion (M1) (Figure 6B); Both the H33A and H35D variants exhibited loss of the M1 ion (Figure 6C and D). By contrast, all three variants retained the Zn ion in the zinc finger-like motif of the β-CASP domain. Our D37A, H33A, and H35D variants lost the ability to digest DNA substrate *in vitro* (Figure 6E), in accord with results with full-length variants (29,33).

Differential scanning fluorimetry (DSF) analyses showed that both H33A and H35D variants are substantially destabilized with ΔT_m_ around -13°C compared to WT Artemis (Supplementary Figure S10). By contrast the D37A variant has similar thermal stability as the WT Artemis, suggesting that it is folded in the presence of a single metal ion in the active site. Asp37 can adopt two conformations and the coordinated zinc ion is present at about 30% occupancy in both of the WT Artemis structures (PDB 6TT5 and 7AF1). It is thus unsurprising that mutation of Asp37 to alanine results in the loss of Zn2.

Histidines 33 and 35 are the first two histidine residues in the HxHxDH motif (M1 binding) in the active site. In the absence of active site metal ions, the loop comprising residues 113–119 moves away from the active site (Figure 6C and D) and a small rearrangement occurs in α8 (residues 348–358). In both the H33A and H35D variants, α8 moves slightly closer towards β14, compared to the WT and D37A variant. Larger differences occur in β-strand E (residues 268–270) and α-helix E (residues 261–267), both located near the zinc finger motif in the β-CASP domain (Figure 6A). In H33A and H35D variants, both β-strand E and α-helix E shifted upward and away from the zinc finger like motif. These conformational changes suggest induced fit during substrate binding. The combined results with the variants thus reveal the importance of the HxHxDH motif and highlight the importance of the di-metal catalytic core in the SNM1 family, not only in directly catalysing hydrolysis, but also likely in conformational changes involved in catalysis (46).

### Identification of small molecule Artemis inhibitors

**Figure 7.**
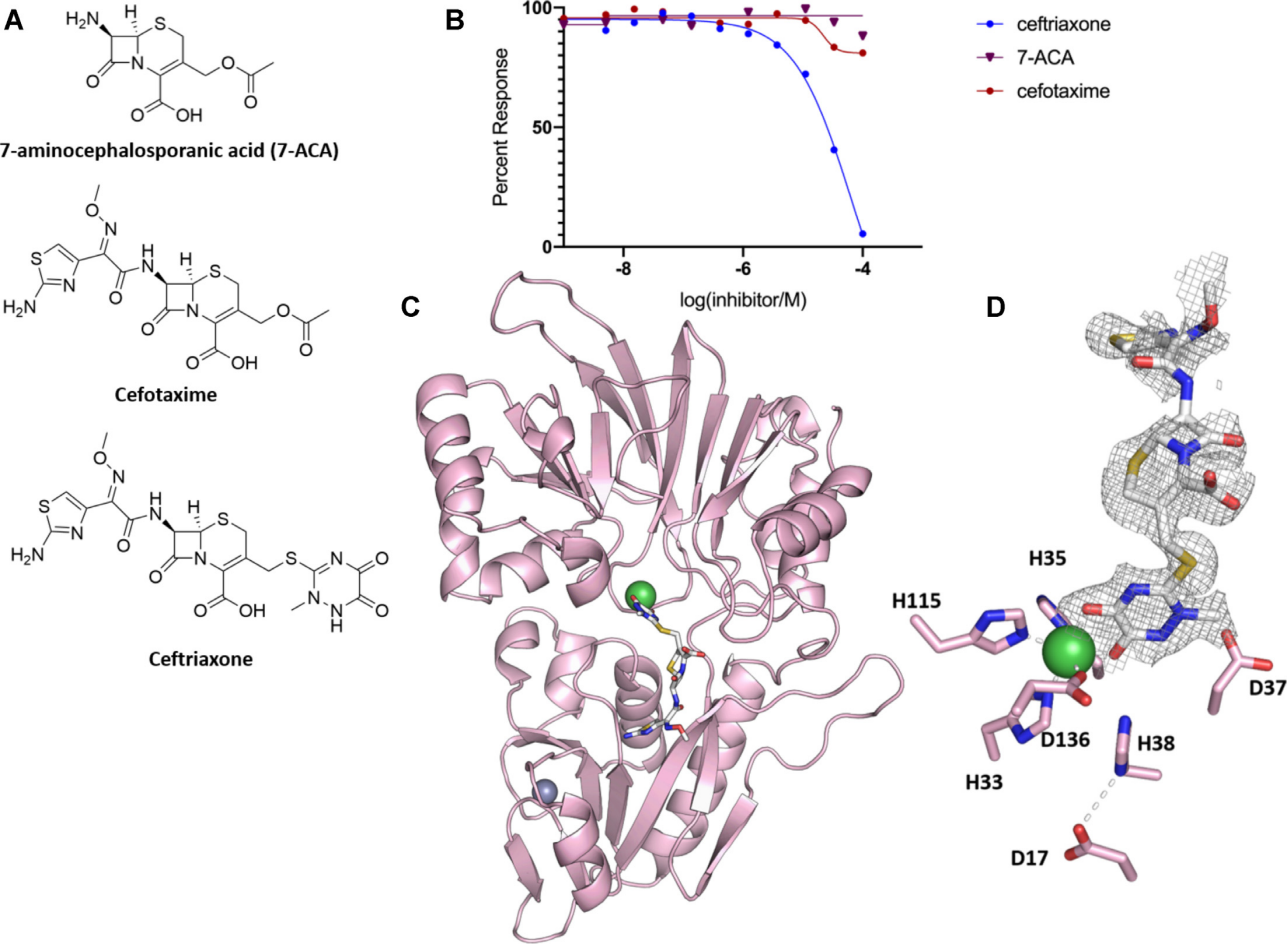
Structural basis of SNM1C/Artemis inhibition by β-lactam antibiotics. (**A**) Structures of selected β-lactam antibiotics. (**B**) Effects of β-lactams on Artemis activity assessed *via* a real-time fluorescence-based nuclease assay. (**C**) Cartoon representation of the structure of truncated Artemis (aa1-361) with ceftriaxone (white) bound at the active site (PDB: 7APV). (**D**) Active site residues with the electron density (*F_o_-F_c_*) contoured map around the modelled ceftriaxone. The map is contoured at the 1.0 σ level and was calculated before ceftriaxone was included in the refinement.

Artemis, along with SNM1A and SNM1B, possess a conserved MBL-fold domain similar to that of the true bacterial MBLs, suggesting that they may be inhibited by β-lactams. Studies on SNM1A/SNM1B, showed that ceftriaxone (Rocephin), a widely used β-lactam (third generation cephalosporin) inhibits SNM1A/SNM1B (63). To investigate if it inhibits Artemis we performed assays with ceftriaxone, cefotaxime, and 7-aminocephalosporanic acid (Figure 7A). The results show that neither cefotaxime nor 7-aminocephalosporanic acid inhibit Artemis, whilst ceftriaxone inhibits with a modest IC_50_ of 65 μM (Figure 7B). We solved a structure of ceftriaxone bound to Artemis (purified by IMAC) at 1.9 Å resolution (Figure 7C) in the space group P1 with one protein molecule in the asymmetric unit. As before, in this structure Artemis possesses the canonical bilobar MBL and β-CASP fold with an active site containing one nickel ion, likely reflecting the purification method.

Ceftriaxone binds to the protein surface in an extended manner making interactions with the active site, towards the β-CASP domain (Figure 7C). There is no evidence for cleavage of the β-lactam, nor of loss of the C-3′ cephalosporin side chain, reactions that can occur during ‘true’ MBL catalysed cephalosporin hydrolysis. Electron density at the active site clearly reveals the ceftriaxone side chain coordinates the M1 site nickel ion replacing two waters (72 and 106) in the uncomplexed structure (Figure 7D). Despite the conservation of key elements of the active site of the MBL fold nucleases and the ‘true’ β−lactam hydrolysing MBLs (64), ceftriaxone, does not interact with the nickel ion *via* its β-lactam carbonyl (as occurs for the true MBLs), but *via* both carbonyl oxygens of the cyclic 1, 2 diamide in its sidechain (Ni–O distances: 2 and 2.2 Å), i.e. it is not positioned for productive β-lactam hydrolysis. The amino-thiazole group (N7) of ceftriaxone forms hydrogen bonds with the side chain of Asn205, while the S1 of the 7-aminocephalosporanic acid core of the compound interacts with the hydroxyl of Tyr212 through an ethylene glycol molecule. The rest of the molecule appears to be flexible. The binding mode of ceftriaxone to Artemis shown in Figure 7C is near identical to that observed for ceftriaxone with SNM1A (PDB: 5NZW) structure (Supplementary Figure S11).

A notable difference between the ceftriaxone-bound Artemis structure and the non-complexed structure is the loss of a metal ion at M2 site, normally coordinated by Asp37, His38 and Asp130 (Figure 7D). Without a metal ion at the M2 site, as in the ceftriaxone bound structure, the Asp37 side chain is positioned away from the active site (Figure 7D), as seen when nickel is bound in the M1 site (Figure 1I).

To investigate the possibility of inhibiting Artemis through binding to the zinc finger motif in the β-CASP domain, we used the fluorescence-based nuclease assay to test compounds known to react with thiol groups present in zinc fingers and which result in zinc ion displacement, i.e. ebselen (65), auranofin (66) and disulfiram (67). Both ebselen and disulfiram inhibit Artemis with IC_50_ values around 8.5 μM and 10.8 μM respectively, whilst auranofin inhibits less potently (IC_50_ 46 μM) (Supplementary Figure S12), indicating additional possible inhibitory strategies.

## DISCUSSION

The DCLRE1C/Artemis gene was first discovered in studies of children with a radiosensitive form of severe combined immunodeficiency disease (RS-SCID) (68). Subsequent work has shown Artemis is a structure-specific endonuclease involved in V(D)J recombination (18,19,69) and the c-NHEJ DNA repair pathway (4,23,70) (2). Our structures of wild-type and catalytic site mutants of Artemis protein show that, like SNM1A and SNM1B/Apollo and the RNA processing enzyme CPSF73, Artemis has a typical α/β-β/α sandwich fold in its MBL domain and has a β-CASP domain, the latter a characteristic of MBL fold nucleases. Both our structures and those of Karim *et al* (37) reveal a unique and conserved structural feature of Artemis in its β-CASP domain that is not reported in other human MBL enzymes, i.e. a conserved (in Artemis orthologues) classical zinc-finger like motif.

Zinc-finger motifs are common in DNA binding proteins such as transcription factors (51,55) and are observed in required and accessory of NHEJ proteins (53). They are reported to both provide stability and enhance substrate selectivity rather than being directly involved in catalysis; we propose that this is likely the case for Artemis. Furthermore, point mutations in His 228 and His 254 (H228N and H254L) have been reported in patients with a SCID phenotype (56). The role of the zinc-finger motif in Artemis is unknown and the subject of ongoing investigations. We have mutated the zinc finger residues (His 228, His 254 Cys 256 and Cys 272), but none of the mutated constructs produced purified protein. Although inconclusive, this may indicate that the Zinc finger is important for the structural integrity or stability of the protein.

The presence of one or two metal ions coordinated by the HxHxDH motif at the Artemis active site is a hallmark of the SNM1 family (11,71); the available evidence implies that metal ion binding at one site (M1 site in standard MBL nomenclature) is stronger than at the other (M2 site). By analogy with studies on the true MBLs, these metal ions are proposed to activate a water / hydroxide ion that act as the nucleophile in the phosphodiester cleavage (72). This hydroxide ion is also present in the crystal structure of CPSF-73 (Figure 1G), along with an adjacent sulphate ion (47), which is proposed to mimic the phosphate group of the pre-mRNA at the cleavage site. Our structure (PDB: 7AF1) suggests that the native metal ion(s) residing in the M1 site of Artemis is zinc, although a nickel ion can also occupy the M1 site depending on the how the protein was purified (PDB: 6TT5). Neither the presence of copurifying Ni ion in the active site, nor the truncation of the C-terminal tail, appear to substantially inhibit the activity of Artemis. Thus, using radio-labelled gel-based nuclease assays, we showed that the truncated Artemis catalytic domain (aa 1–361) with either copurifying Zn or Ni ions (as observed crystallographically in the same preparations) have similar activity with the full-length Artemis construct (aa 1–693). Therefore, it seems likely that nickel ions are able to replace zinc ions in solution; catalysis of MBL fold enzymes, including hydrolytic reactions, with metal ions other than zinc is well-precedented (64,73)

We solved structures of three Artemis variants D37A, H33A and an Omenn syndrome patient mutation, H35D. Using gel-based nuclease assays, we showed that these variants are inactive. Overall, the three variant structures are similar to the WT structure, even though H33A and H35D entirely lack any metal ions in their active site, although zinc was present in the zinc finger. Mutation of Asp37 to alanine results in the loss of the second metal in the catalytic site, likely explaining the loss of activity, although the first metal ion is still present. Note that some MBL fold hydrolases uses two metal ions (e.g. B1 and B3 subfamilies of the true MBLs and RNase J1 from *Bacillus subtilis*) (Supplementary Figure S13A and C) (64,74) whereas others, sometimes with apparently similar active sites, only use one metal ion at the M1 site (e.g. the B2 subfamily of the true MBLs and RNase J2 from *Staphylococcus epidermis*) (64,75) (Supplementary Figure S13B and D). Thus, whilst our results support the importance of having both metals for the nuclease activity by Artemis, subtle features can influence MBL fold enzyme activity (64,71).

Following re-analysis of the Karim *et al.* structure (PDB code 6WO0), we generated a model of a DNA overhang in complex with Artemis that informs on the substrate binding mode. Our model shows that Artemis interacts with its DNA substrate at the interface between the MBL and the β-CASP domains. This interaction is mediated through a combination of polar or positive residues and aromatic residues of Artemis and the DNA substrate (Figure 6). A related interaction was also observed in a cryo-EM structure of the pre-mRNA processing enzyme CPSF-73 with substrate bound, where residues R34, R174, K24 and F176 (CPSF-73 numbering) form direct contacts with the substrate (76). The Artemis DNA bound structure (PDB code 7ABS) contains a 2-base pair region of duplex DNA that makes close contact with the metal centre in the Artemis active site, which could potentially be catalytically relevant (Figure 3). This phosphate ion coordinates the di-metal centre in a similar manner to the AMP molecule bound to the active site of SNM1B/Apollo [Baddock *et al.* 2021, accompanying paper], and the phosphate backbone of the pre-mRNA substrate bound in CPSF-73 (PDB code 6V4X) (76). By analogy with proposals for true MBLs (71,77), the mechanism of SNM1A/B and Artemis catalysed phosphate hydrolysis thus likely involves metal ion mediated reaction of a hydroxide ion with the phosphate and activation of the phosphate by metal ion chelation. However, the precise identity of the *in vivo* relevant SNM1 active site metal ions and whether there are one or two metal ions is uncertain. The true MBLs can be active with two metal ions (B1 and B2 subfamily MBLs) or one metal ion (B2 MBLs); these metal ions are likely zinc ions, but even this has not been unequivocally demonstrated within a human disease context and it is possible that metal ions other than zinc are relevant (77,78). The available evidence is that the SNM1 MBL nucleases employ two (zinc) metal ions, with one (at the M2 site) being less tightly bound than the other (M1 site) – though considerable uncertainty remains as to the *in vivo* relevant metal ions. Binding of a metal ion at the M2 site appears to be promoted by the DNA substrate and it cannot be ruled out that it is (sometimes) delivered by the substrate.

Artemis is the only identified MBL/β-CASP DNA processing enzyme that possesses substantive endonuclease activity. By contrast both SNM1A and SNM1B/Apollo are strictly 5′-phosphate exonucleases (3,11,17). The recent structure of SNM1B/Apollo in complex with two deoxyadenosine monophosphate nucleotides reveals a cluster of residues that form a 5′-phosphate binding pocket, adjacent to the metal centre [Baddock *et al.*, 2021, accompanying paper]. Structural sequence alignments of the three SNM1 proteins shows that these residues are conserved in SNM1A and SNM1B (Supplementary Figure S1). Apart from Ser317, none of these conserved phosphate binding pocket residues are present in Artemis. Instead, the pocket is partially occupied by the Phe318 side chain, which is absent in both SNM1A and SNM1B. Artemis also possesses a longer and more flexible loop connecting β-strands 27 and 28 (Figure 6B), compared to the same loop in SNM1A and SNM1B that make up part of the 5′-phosphate binding pocket. The flexibility of this loop could enable accommodation with different types of DNA structures, such as hairpins, and 5′-overhangs. These differences plausibly explain Artemis’ substrate preferences and its primary activity as a structure-selective endonuclease.

Radiotherapy is a mainstay of cancer therapy; its effectiveness relies on inducing DNA double-strand breaks (DSBs) that contain complex, chemically modified ends that must be processed prior to repair (79,80). The canonical non-homologous end-joining (c-NHEJ) pathway repairs around 80% of DNA double-strand breaks in mammalian cells (22,25). Combining radiation therapy in conjunction with SNM1 inhibitors could selectively radio-sensitize tumours.

Of the three of the β-lactam antibacterial compounds previously shown to inhibit SNM1A (63), we only observed inhibition of Artemis with ceftriaxone. Although the potency of inhibition is moderate (IC_50_ 65 μM), we were able to solve the structure of ceftriaxone in complex with Artemis. Notably, ceftriaxone does not bind with its β-lactam carbonyl located at the active site where it ligates to one zinc (or other metal) ion, but instead binds the single nickel ion in a bidentate manner *via* the carbonyls of the cyclic 1,2 diamide on its C-3′ sidechain (71,81). Studies with the true MBLs have shown that appropriate derivatization of weakly binding molecules can lead to highly potent and selective inhibitors.

In an attempt to inhibit Artemis though its unique zinc-finger like motif, we tested inhibitors with thiol-reactive groups. Ebselen, disulfiram and auranofin have the potential to interact with zinc fingers, including *via* zinc ejection with consequent protein destabilization (66,67,82,83). Both ebselen and auranofin are reported have antimicrobial properties (84) and ebselen is in clinical trials for conditions, ranging from stroke to bipolar disorder (85), and auranofin is used for treatment of rheumatoid arthritis (86). Recent studies have also shown that ebselen inhibits enzymes from SARS-CoV-2, i.e. the main protease (Mpro) and the exonuclease ExoN (nsp14^ExoN^-nsp10) complex (87,88). Disulfiram is a known acetaldehyde dehydrogenase inhibitor used in treatment for alcohol abuse disorder (89). Our results show that both ebselen and disulfiram inhibit Artemis (IC_50_s 8.5 μM and 10.8 μM, respectively), whilst auranofin is less potent (IC_50_ 46 μM).

Studies focussed on inhibiting the MBL fold nucleases are at an early stage compared with work on the true MBLs (78) The structures and assays results presented here provide starting points with established drugs, from which it might be possible to generate selective Artemis inhibitors, either binding at the active site or elsewhere (including the apparently unique zinc finger of Artemis), in order to radio-sensitize cells.

## DATA AVAILABILITY

Coordinates and structure factors are deposited in the Protein Data Bank under codes 6TT5, 7AF1, 7AFS, 7AFU, 7AGI, 7APV and 7ABS.

## Supplementary Material

gkab693_Supplemental_File
