# Supplementary material for: Structural and mechanistic insights into the Artemis endonuclease and strategies for its inhibition
Source: Nucleic Acids Res. 2021 Aug 13;49(16):9310–26. doi: 10.1093/nar/gkab693 (PMC8450076; doi:10.1093/nar/gkab693)
Supplement: gkab693_Supplemental_File [file gkab693_supplemental_file.pdf]

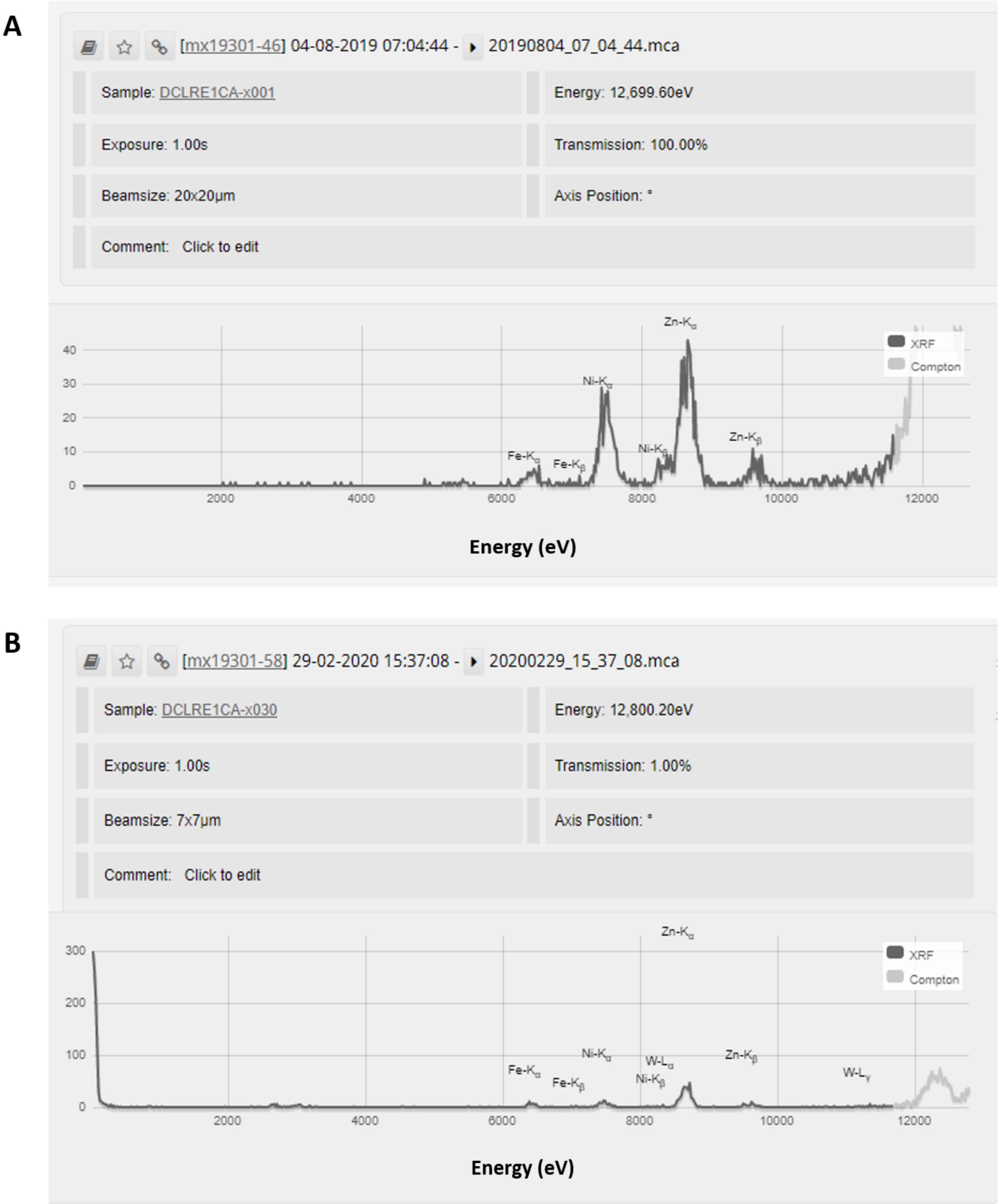

Figure S1: X-ray fluorescence of analysis of metal content in the WT Artemis crystals A: XRF analysis for WT Artemis crystal (PDB: 6TT5) purified using IMAC. The predominant metal species in the crystals are Ni and Zn. **B:** XRF analysis for WT Artemis crystal (PDB: 7AF1) purified without IMAC. Zinc is the predominant metal in this crystal form.

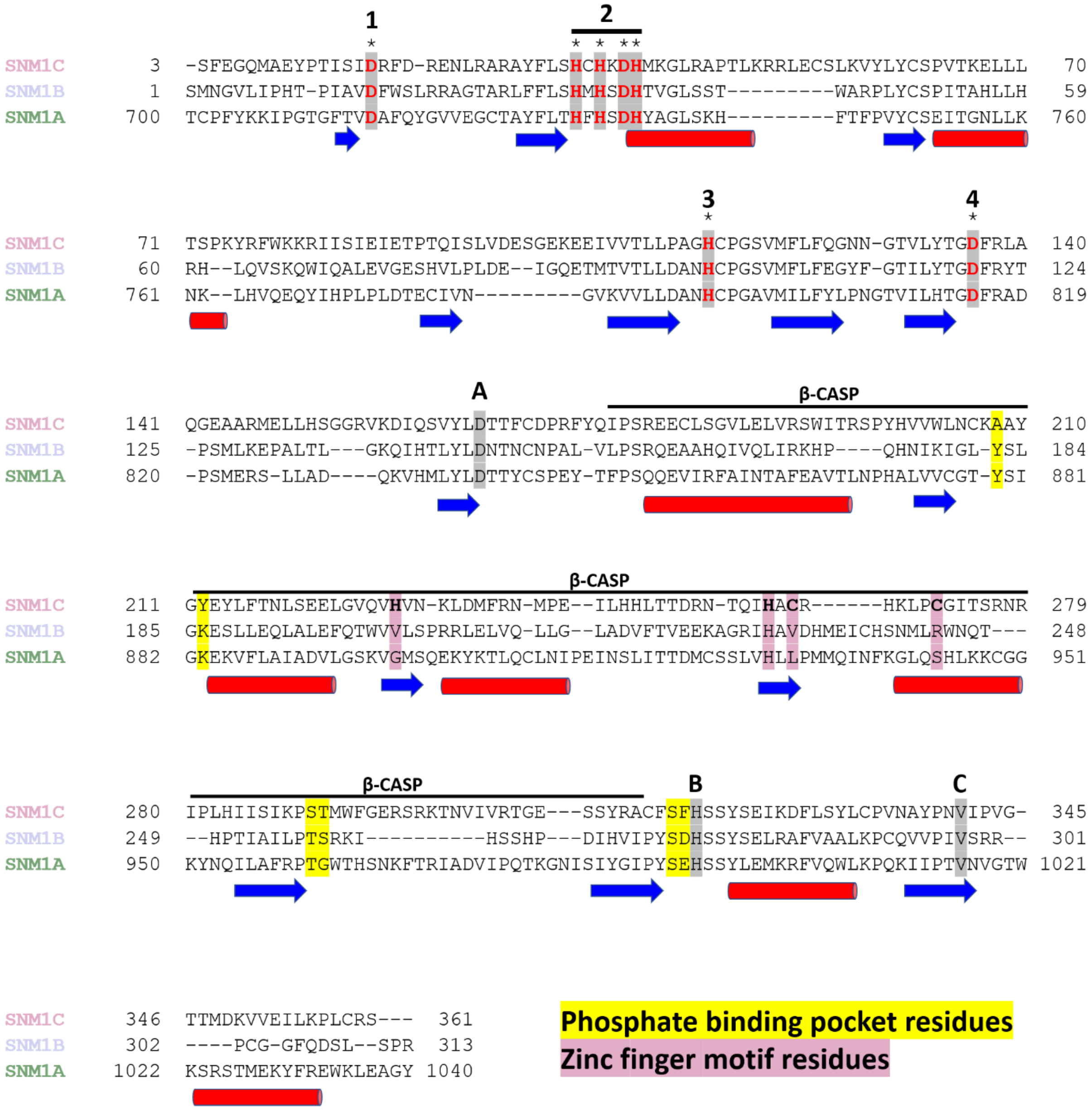

Figure S2: Structurally informed sequence alignments of the human SNM1 nuclease family. The structural alignment was carried out using PROMALS3D [1]. α-helices are drawn as red cylinders and the β-strands as blue arows. The conserved MBL family motifs are labeled as 1–4 and the canonical β-CASP motifs are labeled A–C. The conserved phoshate binding residues in SNM1A and SNM1B are highlighted in yellow. The residues that made up the novel zinc finger like structure are highlighted in pink.

```
                                       1                       2
                                       *                     * * **
Homo_sapiens                 MSSFEGQMAEYPTISIDRFDRENL-RARAYFLSHCHKDHMKGLRAPTLKRRLECSLKVYL   59
Rattus_norvegicus            MSSFQGQMEEYPTISIDRFDRENL-KARAYFLSHCHKDHMKGLRAPSMKRRLECSLKVFL   59
Danio_rerio                  MSSFAGRMKEYPSISLDRFDRENL-HARAYFLSHCHKDHMKGLKGPLLKRKLKFSLTVKL   59
Petromyzon_marinus           MSSFLGKTAEYPSVSIDRFDRDNV-DARAFFLSHCHQDHMVGLYSEALRKKLERSPGVHL   59
Gallus_gallus                MSRFGGRLREYPQLSIDRFDYDNL-RARAYFLSHCHKDHMKGLRAPALRRRLQSSLKVKL   59
Python_bivittatus            MSRFGGVMREYPRLSIDRFDRENL-RARAYFLSHCHKDHMKGLRAPPMKRRLACSLKVHL   59
Xenopus_tropicalis           MSSFMGRMKEYPAISIDRFDRENL-SARAYFLSHCHKDHMKGLRAPFLKRRLQNSLKVHL   59
Chelonia_mydas               MSSFGGRMREYPWLSIDRFDRENL-RARAYFLSHCHKDHMKGLRAPSLKRRLECSLKVHL   59
Amphimedon_queenslandica     MSVFKGSILEYPGLVVDRFNPVNCRRGRAFFLSHCHKDHMSGLDSDELLKVL-KELKIDF   59
                             ** * *   *** : :***:  *   .**:******:*** **  .  : : *  .  : :

                                                                                        3
                                                                                        *
Homo_sapiens                 YCSPVTKELLLTSPKYRFWKKRIISIEIETPTQISLVDE-ASGEKEEIVVTLLPAGHCPG   118
Rattus_norvegicus            YCSPVTKELLLTSPKYKFWENRIIAIEIETPTQVSLVDE-ASGEKEEVVVTLLPAGHCPG   118
Danio_rerio                  YCSYVTKELLLSNPRYAFWEDHIVPLELDSPTSISLIDE-STGETEDVVVTLLSAGHCPG   118
Petromyzon_marinus           YCSPVTRELLLSNWRYRFLERFTVAIEVDTATQISLMDD-KTKQKTDLVVTLLPAGHCPG   118
Gallus_gallus                YCSPVTKELLLTNSKYAFWENHIVALEVETPTQISLVDE-TTGEKEDIEVTLLPAGHCPG   118
Python_bivittatus            YCSPVTKELLLSSPRYKFWENHIVALEVETPTQISLIEE-ASGEKEDIEVTLLPAGHCPG   118
Xenopus_tropicalis           YCSPVTKELLLTNPKYAFWENRMISIEIDTPTQISLVDE-ATGYKEDVVVTLLPAGHCPG   118
Chelonia_mydas               YCSPVTKELLLTSPKYKFWENRIVTLEVETPTQISLLDE-ASGEKEEIVVTLLPAGHCPG   118
Amphimedon_queenslandica     YCSEVTHALLSNDPGFSHLMPYLVSVPVGETISLKLNTFRNEDQVVTVNVTLLPAGHCPG   119
                             *** **: ** ..  : .     : : :    .:.*            : **** ******

                                             4                            A
                                             *
Homo_sapiens                 SVMFLFQGNNGTVLYTGDFRLAQGEAARMELLHS-GGRVKDIQSVYLDTTFCDPRFYQIP   177
Rattus_norvegicus            SVMFLFQGSNGTVLYTGDFRLAKGEVSRMELLHS-GGRVKDIQSVYLDTTFCDPRFYQIP   177
Danio_rerio                  SVMFLFEGAKGTVLYTGDFRLAVGDAARMEYLHS-GDRVKDIQSVYIDTTFFDPKYYQIP   177
Petromyzon_marinus           SVMFLFEGDHGAVLYTGDFRLPRGGVSRMGALHL-AGRIKRIKSIYLDTTFCDPMCYYIP   177
Gallus_gallus                SVMFLFQGENGTVLYTGDFRLAKGEAARMELLHS-GTSVKDIQSVYLDTTFCDPRFYHIP   177
Python_bivittatus            SVMFLFQGENGTVLYTGDFRLARGEVARMELLHS-GSRVKDIQSVYLDTTFYDPKFYQIP   177
Xenopus_tropicalis           SVMFLFQGNSGTVLYTGDFRLAKGEVARMELLHS-GNRVKDIESVYLDTTFCDPKYYQIP   177
Chelonia_mydas               SVMFLFQGQNGTVLYTGDFRLAKGEAARMELLHS-GTRVKDIQSVYLDTTFCDPRFYQIP   177
Amphimedon_queenslandica     SVMFLFQGDAGNVLYTGDFRLSLSDIRGCGPLHTDDGKVIEIKALYLDTTFCHPKSTNII   179
                             ******:*  * *********  .        **      :  *:::*:**** .*    *

                                                    β-CASP
Homo_sapiens                 SREECLSGVLELVRSWITRSPYHVVWLNCKAAYGYEYLFTNLSEELGVQVHVNK--LDMF   235
Rattus_norvegicus            SREECLRGVLELVRSWITRSPKHVVWLNCKAAYGYEYLFTNLSEELGVQVHVDK--LDMF   235
Danio_rerio                  SREACLAGIQQLVQDWICQSPYHVVWLNCKAAYGYEYLFTNLGQEFNSQIHVNS--LDMF   235
Petromyzon_marinus           SREDCLKGILNLVRGWITQSPQHVIWLNCKAAYGYEYLFTHLSREFGQKVHLGY--LEMF   235
Gallus_gallus                SREECLSGILELVRSWTTLSRYHVVWLNCKAAYGYEYLFINLSEELGIKVHVNK--LDMF   235
Python_bivittatus            SREECMKGIMELVRSWVVQSPYHVVWLNCKAAYGYEYLFTNLSEELGIKVHVNK--LDMF   235
Xenopus_tropicalis           SREECLSGILELVRSWITLSPFHVVWLNCKAAYGYEYLFTNLSEEFGAKVHVNK--LDMF   235
Chelonia_mydas               SREECLNGILELVRSWITLSRHHVVWLNCKAAYGYEYLFTNLSEELGVKVHVNK--LDMF   235
Amphimedon_queenslandica     SRDETRDIILKKVKEWLAQGPDNIVRLDCR-SFGYEHILMSLSLQLDTNIHTAGWKIRSY   238
                             **:      : : *: *   .  ::: *:*: ::***:::  *. ::. ::*     :  :

                                                    β-CASP
Homo_sapiens                 RNMPEILHHLTTD-RNTQIHACRHPKAEEYFQWSKLPCGITSRNRIPLHIISIKPSTMWF   294
Rattus_norvegicus            KNMPDILHHLTTD-RNTQIHACRHPKAEEYFQWNKLPCGMASKTKTVLHTISIKPSTMWF   294
Danio_rerio                  KKMPEILCHVTTN-RATQIHACRHPKDEEFFRANRLPCGSTAPDGIPLNIISIKPSTIWF   294
Petromyzon_marinus           QNMPDILDHVTHD-SNTQIHACRIPKDAGICEGRRLPCREKDAKGKPLHMMSIKPSTMWF   294
Gallus_gallus                KNMPEILYHITTD-RYTQIHACRHPKDDDYVRGNRLPCGITCQNGTPLHVISIKPSTMWF   294
Python_bivittatus            KNMPEILYHVTTN-QHTQIHACRHPRDDELFRGNKLPCGITSQNGHQFHVISVKPSTMWF   294
Xenopus_tropicalis           KNMPEILSHITTD-RRTQIHACRHPVNEEFTRANRMPCGMFSDDGIPLHVISIKPSTIWF   294
Chelonia_mydas               NNMPEILYHVTTD-RHTQIHACRHPRDDDYLRGNRLPCGMTSRNGTPLCIISVKPSTMWF   294
Amphimedon_queenslandica     SVLPQVHQCLTEDGNSTRIHACVNKKGTQSV-SGKLPCGATPTKGGTPNILTIKPSAQWF   297
                               :*::   :* :   *:****              ::**              :::***: **

                                   β-CASP               B                C
Homo_sapiens                 GERSRKTNVIVRTGESSYRACFSFHSSYSEIKDFLSYLCPVNAYPNVIPVGTTMDKVVEI   354
Rattus_norvegicus            GERTRKTNVIVRTGESSYRACFSFHSSYSEIKDFLSYICPVNAYPNVIPIGLTVDKVMDF   354
Danio_rerio                  GERTRKTSVVVKMGSSSYRACFSFHSSYLEVKDFLSYICPVNIYPNVIPLGKTVEDLTEL   354
Petromyzon_marinus           TQRSKKTKIIVRTGESSYRACFSFHSSFSEIEDFISYIKPANIFPNVIPKGKTVEDIKEM   354
Gallus_gallus                GERIKKTNVIVRTGESTYRACFSFHSSYSEIMDFLSYIRPVNVYPNVLPVGGSEDKVMEI   354
Python_bivittatus            GERTRKTSVIMRTGMSSYRACFSFHSSYSEIQDFLSHICPENVYPNVIPVGSTEDKVKEN   354
Xenopus_tropicalis           GERTRRTNVIVRTGESSYRACFSFHSSYSEIKDFLGYIKPMNVYANVIPMGKSIEHVEKI   354
Chelonia_mydas               GERTRKTNIVVRTGESSYRACFSFHSSYSEIKDFVGYIRPVNVYPNVIPVGSTADEVVKI   354
Amphimedon_queenslandica     LSNDKPHPSAFFPEFNLFRVLHSMHSSYSEIIEVVSYLCPVSIVPCVIPYTLGDTSLVDI   357
                              .. :      .     . :*. .*:***: *: :.:.:: * .    *:*        : .
```

Figure S3: Sequence alignments of the catalytic core of DCLRE1C/SNM1C/Artemis protein showing conservation of the zinc finger like motif across diferent species from human to sea sponge. The alignment was carried out using Clustal Omega (EMBL-EBI).The conserved MBL family motifs are labeled as 1–4 and the cannocial β-CASP motifs are labeled A–C. The conserved residues that made up the novel zinc finger like structure are highlighted in green.

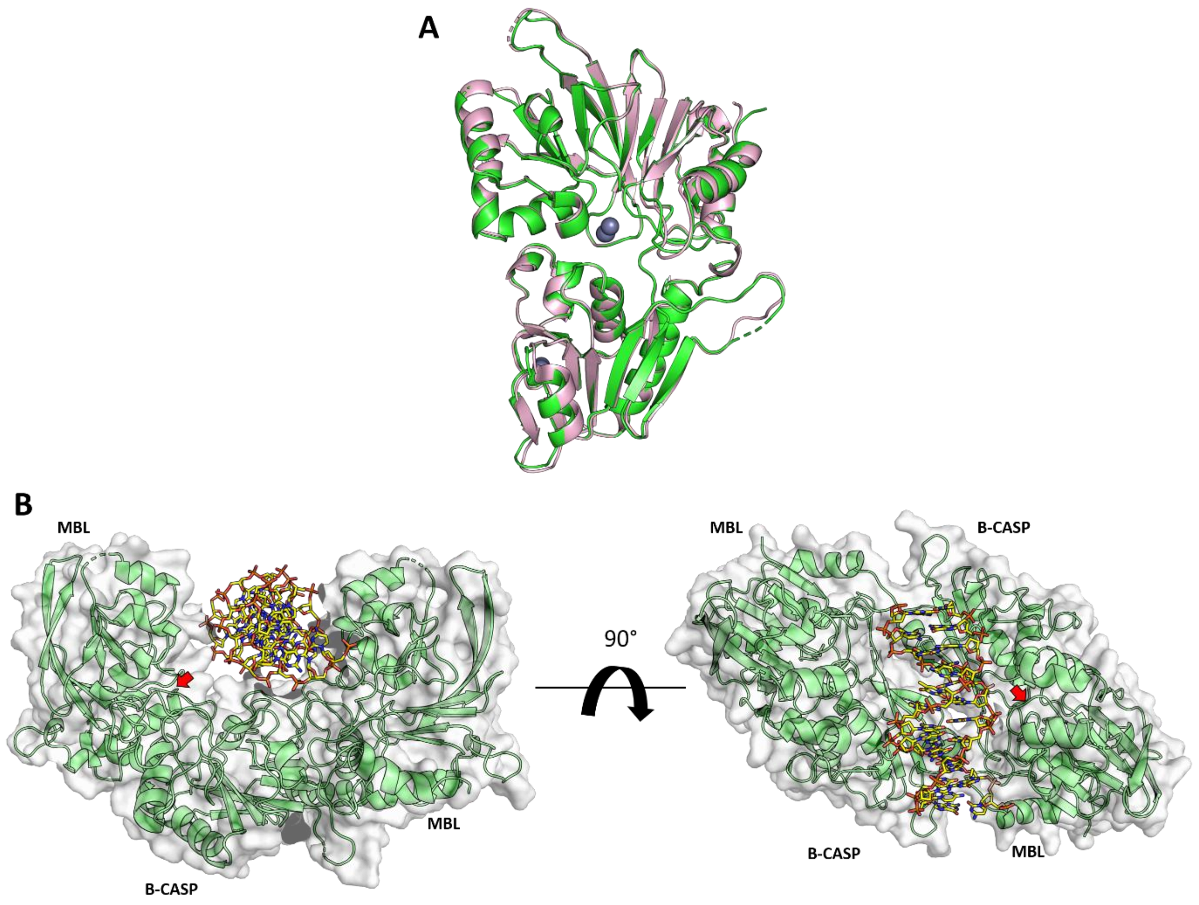

Figure S4: Features of the Artemis structure reported by Karim *et. al* (PDB: 6WNL). A: Overlay of our Artemis structure (PDB: 7AF1) in pink and 6WNL in green. **B:** Re-anaylsis of 6WNL with a model of DNA hairpin (yellow) refined in the solvent chanel. The active sites of both Artemis molecules are indicated with red arrows.

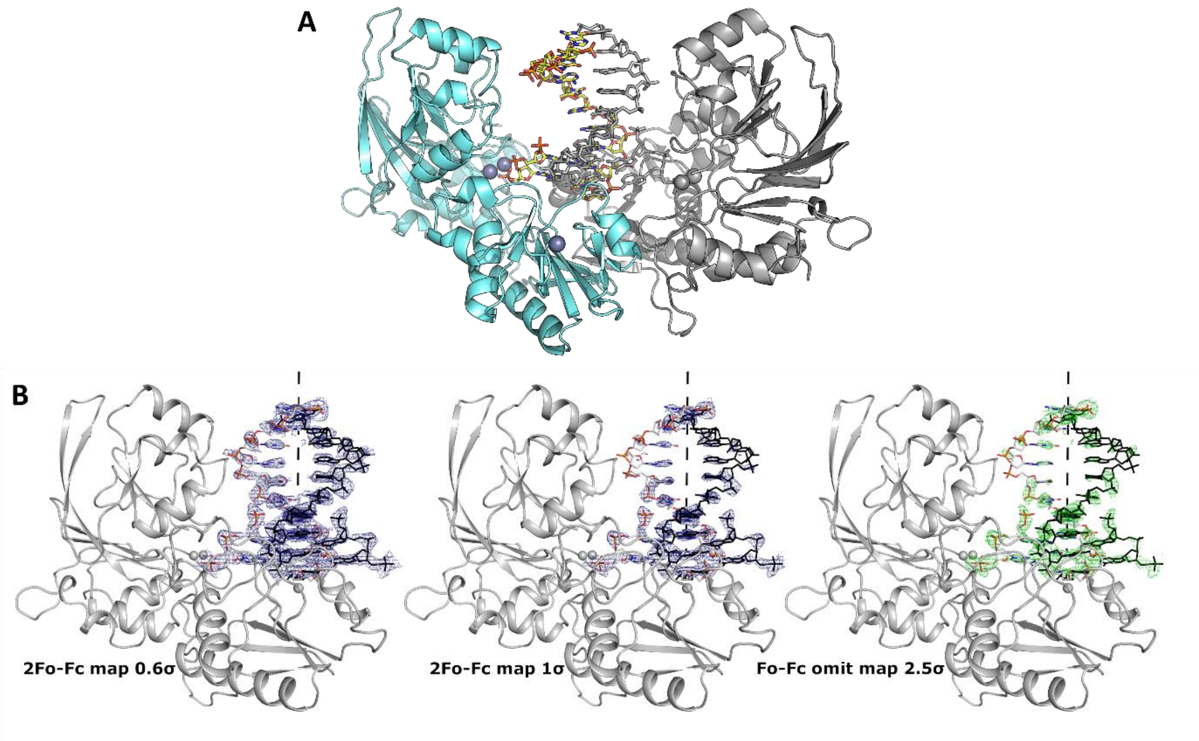

Figure S5: Overall strcuture of DNA bound Artemis (PDB: 7ABS). A: The structure of DNA bound Artemis showing a two-fold symmetry axis, where the symmetry related molecule is shown in gray. **B:** View of the structure of the Artemis DNA complex with a diferent map contours. The $2F_o$-F_c maps are presented in in grey mesh for 0.6 and 1.0 σ, while the F_o-F_c maps is in green at 2.5 σ.

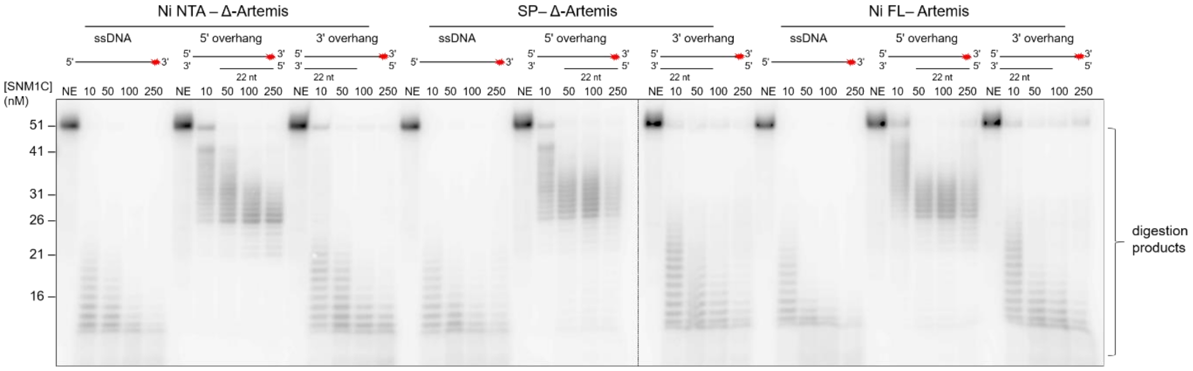

Figure S6: Nuclease activities of WT Artemis. Full-length and truncated Artemis constructs purified in two ways were used. Increasing amounts of enzyme (concentrations as indicated) were incubated with 10 nM of either ssDNA, 5’ overhang, or 3’ overhang DNA substrate for 45 min at 37 °C. Reaction products were subsequently analysed by 20% denaturing PAGE. The sizes (in nucleotides) of the marker oligonucleotides are indicated on the left-hand side of the corresponding bands.

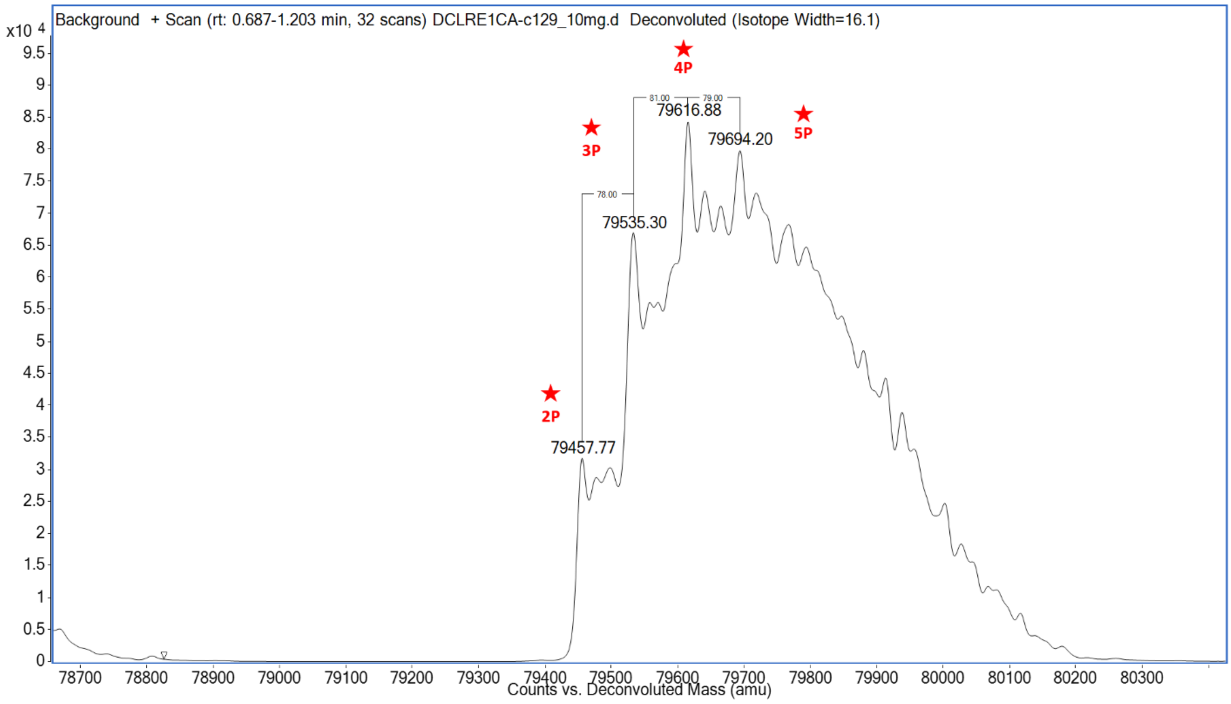

Figure S7: Intact mass analysis of the full length Artemis (aa 1–693) showing evidence for five phosphorylation states (red stars).

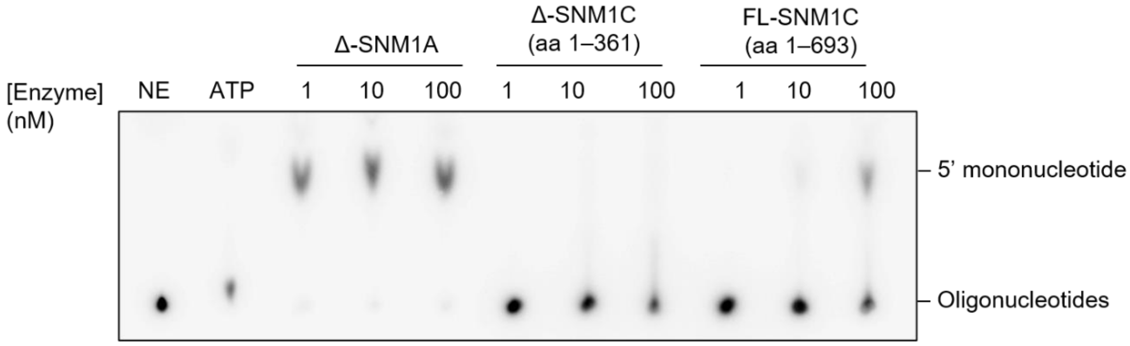

Figure S8: Full-length Artemis exhibits weak exonuclease activity, and Δ-Artemis negligible exonuclease activity. Exonuclease activity was examined by thin layer chromatography with increasing enzyme concentrations (as indicated) and 10 nM of 5'-labelled ssDNA (50 nt) for 20 minutes at 37 °C. The samples were spotted on PEI-cellulose TLC sheets and developed with 400 mM phosphate buffer, pH 4.3 as the liquid phase. ATP and oligonucleotides remain near the origin, while the 5'-mononucleotide released by exonuclease activity migrates with the mobile phase.

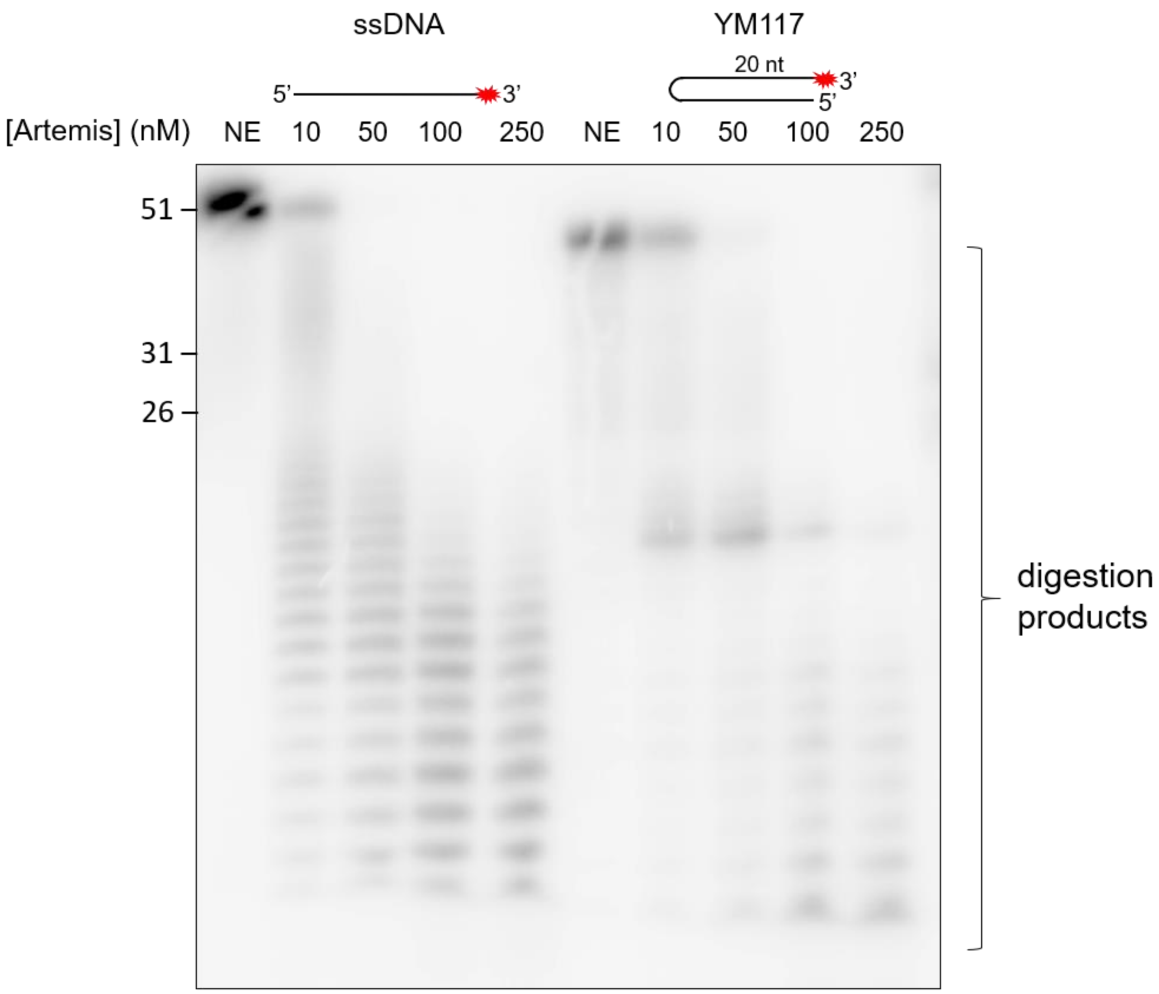

Figure S9: WT Artemis exhibits hairpin opening activity. Increasing amounts (from 0 to 250 nM) of WT Artemis were incubated with 10 nM of 51 nucleotide ssDNA substrate or a duplex hairpin substrate (YM117 from Ma *et al.,* 2002) for 45 min at 37°C. Reaction products were subsequently analysed by 20% denaturing PAGE. The sizes (in nucleotides) of the marker oligonucleotides are indicated on the left-hand side of the corresponding bands.

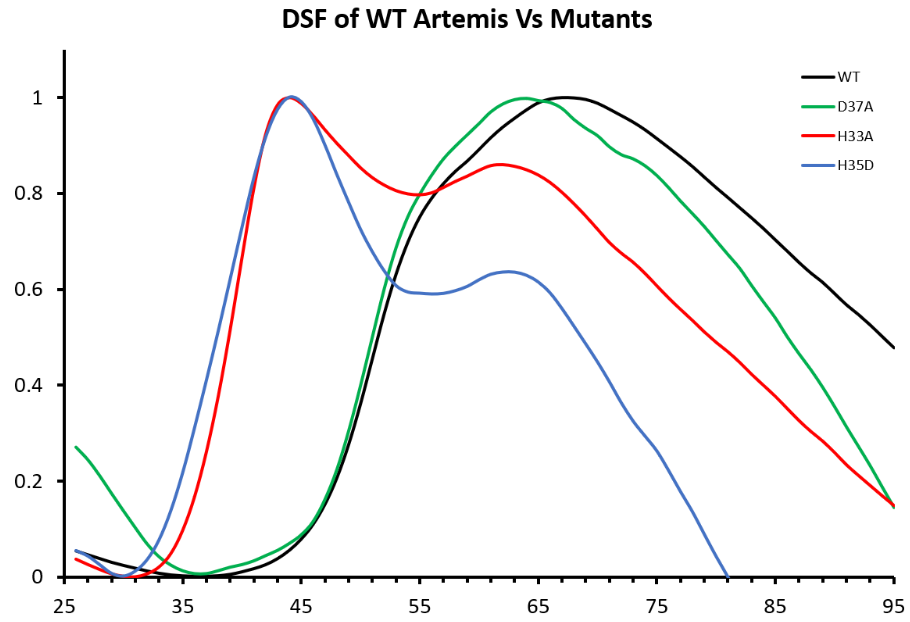

	Tm
**WT**	52
**H35D**	38
**H33A**	39
**D37A**	51

Figure S10: Thermal stability of Artemis WT and mutants assayed using differential scanning fluorimetry (DSF). The H35D (blue) and H33A (red) variants exhibit a 13–14°C lower melting temperature compared to WT protein, whilst the D37A variant (green) has a very similar denaturation curve. The melting temperature of the proteins are presented in the right hand side table.

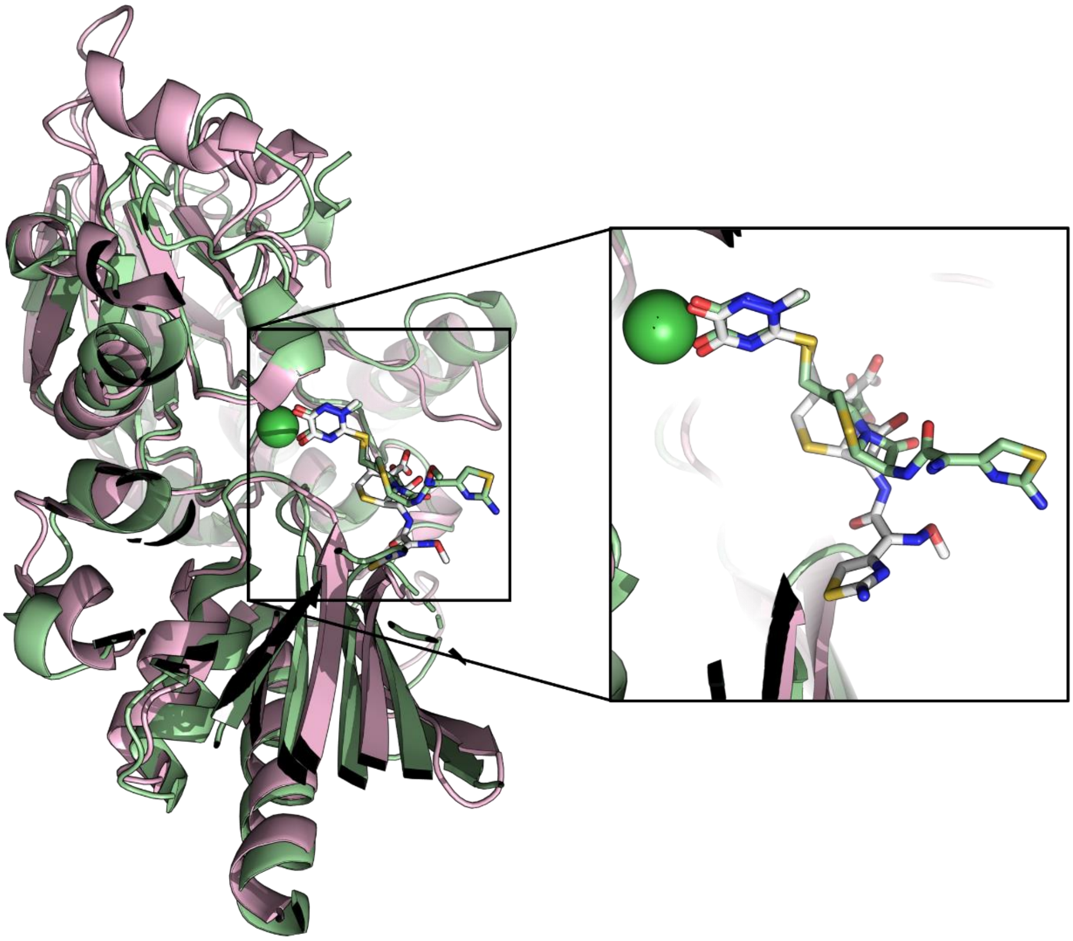

Figure S11: Structures of SNM1A and SNM1C (Artemis) in complex with ceftriaxone. The figure shows an overlay of SNM1A in green (PDB: 5NZW) and SNM1C (PDB: 7APV) in pink, both in complex with ceftriaxone. The inset shows the binding mode of ceftriaxone to the metal centre through its cyclic 1, 2 diamide functional group.

A

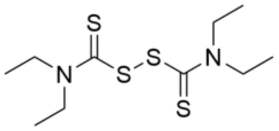

Disulfiram IC_{50} = 10.8 µM

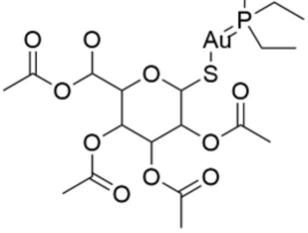

Auranofin IC_{50} = 46.0 µM

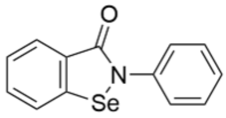

Ebselen IC_{50} = 8.5 µM

B

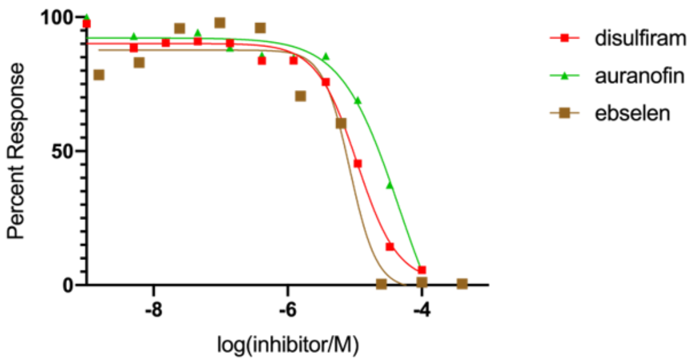

Figure S12: Inhibition of truncated Artemis (aa 1–361) by compounds containing thio-reactive groups A: Structures of disulfiram, auranofin, and ebselen, and their coresponding IC_{50} values. **B:** Inhibition curves of the three compounds against Artemis obtained using the real-time fluorescence-based nuclease assay.

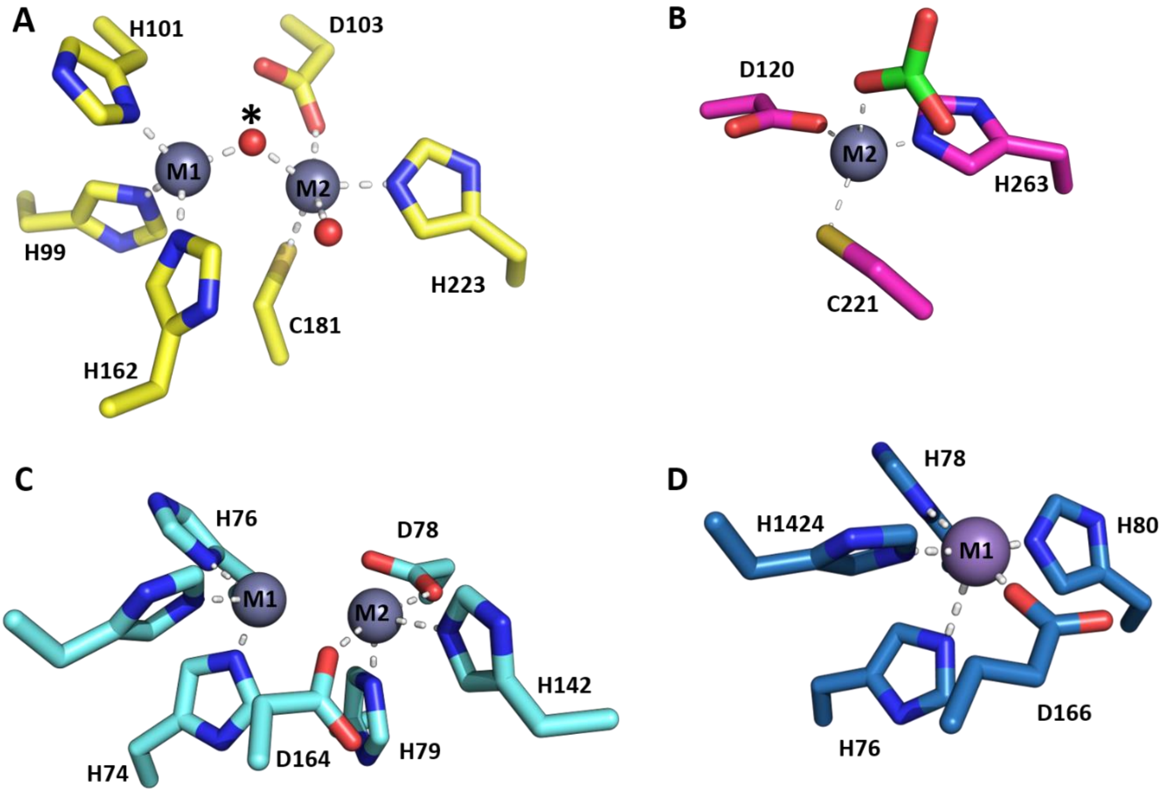

Figure S13: Active site views of bacterial MBL and bacterial MBL/β-CASP enzymes A: Active site view of a bacterial metallo-β-lactamase (subfamily B1) from *Bacteroides fragilis* (PDB: 1ZNB) showing coordination of two zinc ions (in grey). A water molecule positioned (asterisk*) between the two metals is the proposed nucleophile for the hydrolytic reaction. **B:** The active site view of the Carbapenemase CphA from *Aeromonas Hydrophyla*, which is from metallo-β-lactamase subfamily B2 (PDB: 1X8G) has only a single active site zinc ion (in grey) with carbonate ion (green) complexed **C:** The active site structure of RNase J1 from *Bacillus subtilis* (PDB: 3ZQ4) with two zinc ions bound (in grey). **D:** The active site of RNase J2 from *Staphylococcus epidermidis* (PDB: 6K6W) contains a single manganese ion (in purple).

Table S1: List of DNA oligonucleotide sequences used to generate DNA substrates.

(A) the top panel indicates each oligonucleotide sequence as numbered. (B) Scematic representation of the substrates used in nuclease assays. The single-stranded oligos indicated by the codes were annealed as described in the Materials and Methods. A red asterisk indicates location of a radiolabel; a yellow asterisk, the position of FITC; and a dark blue circle, the location of BHQ-1

A

Code	DNA oligonucleotide sequence
**1A**	5’P-ATAAATATTTTTTATTAATAATAGATCACCTTTCTTTCTCTTCTCCCCTT-OH3’
**1B**	5’OH-ATAAATATTTTTTATTAATAATAGATCACCTTTCTTTCTCTTCTCCCCTT-OH3’
**1C**	5’biotin-ATAAATATTTTTTATTAATAATAGATCACCTTTCTTTCTCTTCTCCCCTT-OH3’
**2**	5’OH-AAGGGGAGAAGAGAAAGAAAGGTGATCTATTATTAATAAAAAATATTTAT-OH3’
**3**	5’OH-AAGGGGAGAAGAGAAAGAAAGG-OH3’
**4**	5’OH-ATTATTAATAAAAAATATTTAT-OH3’
**5**	5’OH-TTCCCCTCCTCTCCTTCCTTCCTGATCTATTATTAATAAAAAATATTTAT-OH3’
**6**	5’OH-AAGGGGAGAAGAGAAAGAAAGG-OH3’
**7**	5′OH-GATTACTACGGTAGTAGCTACGTAGCTCTACCGTAGTAAT-OH3′
**8**	5’OH-[FITC]TAATTAATAATAGATCACCT[BHQ1]-OH3’

B

3’ labelled substrates (α-^{32}P-dATP)			
**Annealed DNA Sequences**	**Substrate Structure**	**Description**	**Figure(s)**
1A*	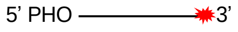	Single-stranded 51 nt DNA with a 5′ phosphate	7, 9, Suppl. 6
1B*	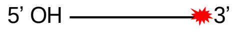	Single-stranded 51 nt DNA with a 5′ hydroxyl	7
1C*	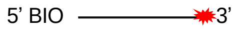	Single-stranded 51 nt DNA with a 5′ biotin	7
1A* + 2	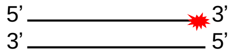	dsDNA	7
1A* + 3	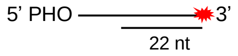	5′ overhang with the labelled strand bearing a 5′ phosphate	7, Suppl. 6
1B* + 3	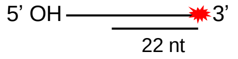	5′ overhang with the labelled strand bearing a 5′ hydroxyl	7
1C* + 3	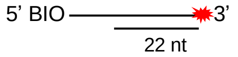	5′ overhang with the labelled strand bearing a 5′ biotin	7
1A* + 4	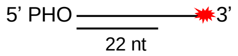	3′ overhang with the labelled strand bearing a 5′ phosphate	7, Suppl. 6
1B* + 4	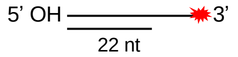	3′ overhang with the labelled strand bearing a 5′ hydroxyl	7
1C* + 4	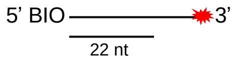	3′ overhang with the labelled strand bearing a 5′ biotin	7
1A* + 5	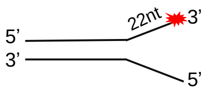	Splayed arm	7
1A* + 5 + 3	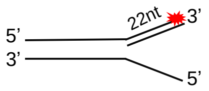	Leading strand flap	7
1A* + 5 + 6	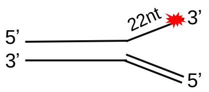	Lagging strand flap	7
1A* + 3 + 5 + 6	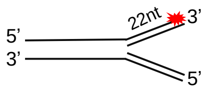	Replication fork	7
7*	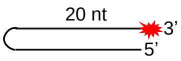	20 nt hairpin substrate (YM117 from Ma *et al.*, 2002)	Suppl. 9
5’ labelled substrates (γ-^{32}P-dATP)			
1B*	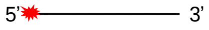	Single stranded 50 nt DNA labelled with a γ-^{32}P-dATP	Suppl. 8
Fluorescently labelled substrates (used for inhibitor screening)			
8	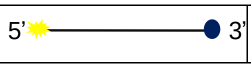	20 nt ssDNA substrate with a 5′ FITC and a 3′ BHQ-1	11, Suppl.12
